# Along‐tract statistics of neurite orientation dispersion and density imaging diffusion metrics to enhance MR tractography quantitative analysis in healthy controls and in patients with brain tumors

**DOI:** 10.1002/hbm.25291

**Published:** 2020-12-04

**Authors:** Valentina Pieri, Francesco Sanvito, Marco Riva, Alessandro Petrini, Paola M. V. Rancoita, Sara Cirillo, Antonella Iadanza, Lorenzo Bello, Antonella Castellano, Andrea Falini

**Affiliations:** ^1^ Vita‐Salute San Raffaele University Milan Italy; ^2^ Neuroradiology Unit and CERMAC, IRCCS San Raffaele Scientific Institute Milan Italy; ^3^ Department of Medical Biotechnology and Translational Medicine Università degli Studi di Milano Milan Italy; ^4^ Neurosurgical Oncology Unit Humanitas Clinical and Research Center – IRCCS Milan Italy; ^5^ Department of Computer Science Università degli Studi di Milano Milan Italy; ^6^ University Centre for Statistics in the Biomedical Sciences, Vita‐Salute San Raffaele University Milan Italy; ^7^ Department of Oncology and Hemato‐Oncology Università degli Studi di Milano Milan Italy

**Keywords:** along‐tract profile, brain tumors, diffusion tensor imaging, HARDI MR tractography, neurite orientation dispersion and density imaging

## Abstract

Along‐tract statistics analysis enables the extraction of quantitative diffusion metrics along specific white matter fiber tracts. Besides quantitative metrics derived from classical diffusion tensor imaging (DTI), such as fractional anisotropy and diffusivities, new parameters reflecting the relative contribution of different diffusion compartments in the tissue can be estimated through advanced diffusion MRI methods as neurite orientation dispersion and density imaging (NODDI), leading to a more specific microstructural characterization. In this study, we extracted both DTI‐ and NODDI‐derived quantitative microstructural diffusion metrics along the most eloquent fiber tracts in 15 healthy subjects and in 22 patients with brain tumors. We obtained a robust intraprotocol reference database of normative along‐tract microstructural metrics, and their corresponding plots, from healthy fiber tracts. Each diffusion metric of individual patient's fiber tract was then plotted and statistically compared to the normative profile of the corresponding metric from the healthy fiber tracts. NODDI‐derived metrics appeared to account for the pathological microstructural changes of the peritumoral tissue more accurately than DTI‐derived ones. This approach may be useful for future studies that may compare healthy subjects to patients diagnosed with other pathological conditions.

## Graphical abstract

Neurite orientation dispersion and density imaging (NODDI)‐ and diffusion tensor imaging (DTI)‐metrics extracted along white matter fascicles reflect tract‐specific microstructure and integrity. Along‐tract metrics from brain tumor patients were compared to a intrastudy reference database in order to highlight pathological white matter alterations in the peritumoral fascicles. NODDI‐metrics reflected pathological microstructural changes of the peritumoral tissue more accurately than DTI‐derived ones, and are more useful to disentangle the different contributions of tissue compartments to the diffusion signal.

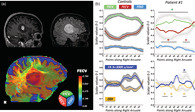

**Abbreviations**
AD: axial diffusivity
AF: arcuate fasciculus
CING: cingulum
CST: corticospinal tract
DTI: diffusion tensor imaging
FAT: frontal aslant tract
FECV: fraction of extracellular volume
FICV: fraction of intracellular volume
FISO: fraction of isotropic diffusion
HARDI: high angular resolution diffusion imaging
IFOF: inferior fronto‐occipital fasciculus
MD: mean diffusivity
NODDI: neurite orientation dispersion and density imaging
ODI: orientation dispersion index
OR: optic radiation
RD: radial diffusivity
UF: uncinate fasciculus
WM: white matter

## 1 INTRODUCTION

Diffusion MR tractography depicts white matter (WM) bundles by quantifying the displacement of water molecules within tissue over time and correlating its directionality with the microstructural integrity of myelinated fibers, both in normal and pathological conditions (Catani & Thiebaut de Schotten, 2008; Jellison et al., 2004). Novel approaches have been recently developed to measure and analyze diffusion MRI (dMRI) data along WM tracts, quantifying within‐tract statistical variability of classical diffusion tensor imaging (DTI)‐derived metrics such as mean, axial, and radial diffusivities (MD, AD, RD) and fractional anisotropy (FA) (Colby et al., 2012; O'Donnell, Westin, & Golby, 2009; Yeatman, Richie‐Halford, Smith, Keshavan, & Rokem, 2018). Along‐tract analyses allow to precisely localize variations in diffusivity; conversely, the analysis of the average of metrics over all voxels of the tract flattens subtle changes by considering the bundle as a whole. DTI‐derived metrics are currently used as surrogate measures of tissue microstructure, although they are sensitive to WM deranging but inherently nonspecific. In fact, alterations in the estimates derived from the tensor may depend from several possible biological mechanisms underlying WM microstructural modifications, including demyelination, reduction of axonal density, and increase of neurite orientation dispersion (Adluru et al., 2014; Alexander, Dyrby, Nilsson, & Zhang, 2017; Szczepankiewicz et al., 2015). More sophisticated methods have been proposed to address DTI limitations and disentangle the different microstructural contributions to FA. Among them, the neurite orientation dispersion and density imaging (NODDI) model enables a more detailed tissue characterization than the classical DTI metrics, and it is specific for brain tissue. NODDI was developed to quantify the microstructural complexity and orientation dispersion of dendrites and axons in vivo, by estimating the relative contribution of three different diffusion compartments to the total diffusion signal in each voxel: intracellular volume, extracellular volume and free fluid volume (Zhang, Schneider, Wheeler‐Kingshott, & Alexander, 2012). The NODDI model has been deployed to examine physiological alteration in neurites throughout aging (Nazeri et al., 2015), and in neurological disorders (Rae et al., 2017; Winston et al., 2014). A promising application of NODDI diffusion model is the microstructural characterization of peritumoral tissue in patients with brain malignancies, where conventional MRI and DTI are not capable of discriminating tumor infiltration from vasogenic edema. Recent studies demonstrated the feasibility of studying glioma patients with a clinically compatible multicompartmental dMRI acquisitions and subsequent NODDI analysis (Masjoodi, Hashemi, Oghabian, & Sharifi, 2018; Wen et al., 2015) for the characterization of peritumoral tissue, suggesting that NODDI can be employed to distinguish the extraneurite compartment from edema. To our knowledge, NODDI‐derived quantitative microstructural diffusion metrics have never been extracted along WM tracts, and the combination of tractography and NODDI in depicting peritumoral modifications has not been assessed yet.

Advanced methods for MR tractography incorporate acquisition schemes allowing to perform both NODDI analysis and high angular resolution diffusion imaging (HARDI) tractography, such as q‐ball tractography (Berman et al., 2008) or constrained spherical deconvolution (Tournier, Calamante, Gadian, & Connelly, 2004), which were already proven applicable to brain tumor patients (Becker et al., 2019; Caverzasi et al., 2015; Mormina et al., 2016; Sanvito et al., 2020), and demonstrated higher accuracy than classic DTI‐tractography in the clinical setting (Bucci et al., 2013). Tractography is extensively employed in the presurgical workup of patients with brain tumors, to noninvasively identify the trajectories of eloquent WM tracts located in the proximity or inside the lesions, that should be spared by the surgeons to avoid serious impairment in patient's motor, cognitive, or visual functions (Castellano, Cirillo, Bello, Riva, & Falini, 2017; Riva et al., 2011). Fiber bundles can be incorporated into imaging to define their relationship with the tumor, and may provide pathways for the spread of disease, thus showing different diffusion characteristics when pathologically infiltrated (Castellano et al., 2012; Caverzasi et al., 2015).

Given the clinical relevance of HARDI Tractography advanced techniques, the increasing significance of quantitative along‐tract analyses, and the limitations of DTI‐derived metrics, the main aim of our work was to combine the specificity of NODDI and the accuracy of along‐tract statistics. This approach was employed to explore the microstructural WM tract variability in healthy volunteers, and the tumor‐induced WM abnormalities in patients with brain neoplasms. In this study, for the first time, we propose a systematic quantification of both DTI‐ and NODDI‐derived diffusion metrics along the tract profile of the most eloquent human WM bundles reconstructed by a q‐ball algorithm, bilaterally. The combination of along‐tract statistics and the NODDI model is hereby investigated, to assess whether along‐tract NODDI metrics reflect WM alterations more accurately than along‐tract DTI and localize them in specific WM tracts more precisely than NODDI‐derived maps alone. A reproducible database displaying values from healthy subjects has been constructed and used as intraprotocol reference, in order to perform statistical comparisons between controls and pathological groups, as well as single‐subject analyses.

## 2 MATERIALS AND METHODS

### 2.1 Subjects

Healthy controls' cohort included 15 healthy subjects (9 men, 6 women; mean age, 38 years; range, 24–66 years), who had no history of neurological disorders, and no brain abnormalities on MRI scans. Patients' cohort included 22 subjects with brain tumors (11 men, 11 women; mean age, 47 years; range, 20–78 years), whose histopathological and molecular data are summarized in Table 1. All subjects were enrolled in the EDEN2020 project, were right‐handed as determined by the Edinburgh Handedness Inventory test (Oldfield, 1971) and provided written informed consent to have their data used for research purpose. All procedures were approved by the OSR Institutional Ethics Committee.

**TABLE 1:** Patients' sample characteristics

Variable	Value
Age	Mean age 47 years (range 20–78 years)
Sex	
Male	11
Female	11
Side of tumor (left/right)	
Left (L)	10
Right (R)	12
Histopathology	
Gliomas	15 (6L/9R)^a^
Lower‐grade glioma (WHO II‐III)	8
IDH1/2 mutation and 1p19q codeletion	1 (R)
IDH1/2 mutation and NO 1p19q codeletion	5 (1L/4R)
IDH1/2 wild type	2 (L)
Glioblastoma (WHO IV)	7
IDH1/2 mutation	1 (L)
IDH1/2 wild type	6 (2L/4R)
Gliomas (neuroradiological diagnosis)	4 (L)
Metastasis	1 (R, lung cancer)
Meningiomas	2 (R)

^a^ Number of lesions for each side are reported in parentheses.

### 2.2 MRI acquisition protocol

Datasets were acquired on a 3 T Ingenia CX scanner (Philips Healthcare, Best, The Netherlands), using a 32‐channel head coil. NODDI protocol consisted in a two‐shell acquisition based on axial single‐shot spin‐echo echo planar imaging with an anterior–posterior phase‐encoding direction that included:

- HARDI acquisition: 60 diffusion‐weighted volumes (diffusion gradients were applied along 60 noncollinear directions; *b*‐value, 3,000 s/mm^2^).
- DTI acquisition: 35 diffusion‐weighted volumes (diffusion gradients were applied along 35 noncollinear directions; b‐value, 711 s/mm^2^).
- 11 “B0” volumes without diffusion‐weighting (*b*‐value, 0 s/mm^2^), whose acquisition was placed in between the aforementioned diffusion‐weighted volumes.

Finally, a “reverse B0” volume without diffusion‐weighting was acquired (*b*‐value, 0 s/mm^2^), which shared with the NODDI sequence all of the geometrical features but the phase‐encoding direction, that was posterior–anterior, in order to allow for the subsequent correction of susceptibility artifacts. Conventional MRI protocol included an axial 3D fluid attenuated inversion recovery (3D‐FLAIR) (TR/TE/TI 9,000/290/2,500 ms; flip angle, 40°; 204 slices; thickness, 0.7/−0.5 mm gap; matrix, 204 × 197; SENSE reduction factor R = 2; acquisition time 7 min 30 s), and a sagittal 3D T1‐weighted sequence (TR/TE 12/5.9 ms; flip angle, 8°; 236 slices; thickness, 0.8/0 mm gap; matrix, 320 × 299; SENSE reduction factor R = 2; acquisition time 5 min 19 s) that was acquired after contrast agent administration in patients, whereas contrast agent was not administered to the healthy controls.

### 2.3 NODDI preprocessing

All NODDI volumes were corrected for movement and eddy‐current distortions, using the “eddy” tool of FMRIB Software Library (FSL, University of Oxford, https://fsl.fmrib.ox.ac.uk/fsl/). The reverse B0 volume was then used to correct the datasets for susceptibility‐induced artifacts, by applying FSL built‐in “top‐up” tool.

### 2.4 Generation of NODDI, DTI, and HARDI maps

**FIGURE 1:**
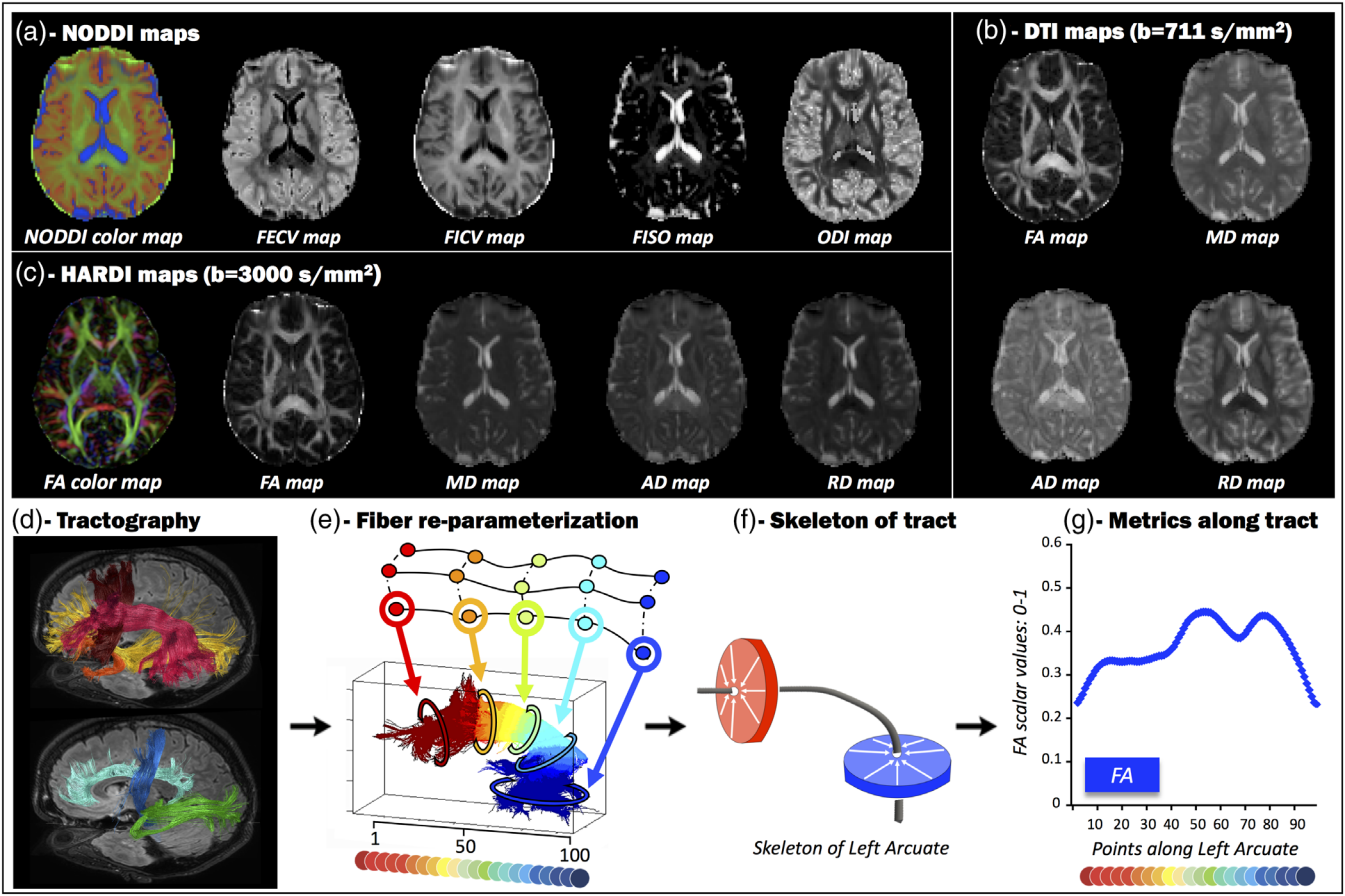
Working pipeline. Representation of the steps necessary for the along‐tract extraction of quantitative diffusion metrics. (a) Computation of neurite orientation dispersion and density imaging (NODDI) maps: voxel fraction of Gaussian anisotropic diffusion (extracellular volume fraction: FECV), voxel fraction of non‐Gaussian anisotropic diffusion (intracellular: FICV), voxel fraction of isotropic Gaussian diffusion (FISO), and orientation dispersion index (ODI) maps. The NODDI compartment maps were combined into a single 4‐dimensional volume visualized as RGB image (red for FECV, green for FICV, and blue for FISO). (b) Computation of diffusion tensor imaging (DTI) maps at *b*‐value 711 s/mm^2^: fractional anisotropy (FA), mean diffusivity (MD), axial diffusivity (AD), and radial diffusivity (RD). (c) Computation of high angular resolution diffusion imaging (HARDI) maps at *b*‐value 3,000 s/mm^2^: FA, MD, AD, and RD. Color‐coded FA maps display fibers with craniocaudal direction in blue, fibers with anteroposterior direction in green, and fibers with mediolateral direction in red. (d) Tractography reconstructions based on a q‐ball residual bootstrap algorithm. Red = arcuate fascicle (AF), brown = frontal aslant tract (FAT), yellow = inferior fronto‐occipital fascicle (IFOF), orange = uncinate fascicle (UF), cyan = cingulum (CING), blue = corticospinal tract (CST), green = optic radiation (OR). (e) Fiber reparameterization computed by the MATLAB Along‐Tract Stats toolbox. Details of each tract are reported in Supplementary Figure 1. (f) Skeleton of tract obtained by averaging the spatial coordinates of all streamline vertices and collapsing them in a single point. (g) Example of diffusion metric plotted in line graph: FA along left AF

Once preprocessing was completed, the Watson‐NODDI model was fitted to the two‐shell dMRI datasets (NODDI acquisition: 60 directions at *b*‐value 3,000 s/mm^2^, 35 directions at *b*‐value 711 s/mm^2^, 11 B0 volumes) using the MATLAB NODDI toolbox (http://mig.cs.ucl.ac.uk/Tutorial.NODDImatlab) to extract the following NODDI maps (Figure 1a): voxel fraction of Gaussian anisotropic diffusion (extracellular volume fraction [FECV]), voxel fraction of non‐Gaussian anisotropic diffusion (intracellular volume fraction [FICV]), voxel fraction of isotropic Gaussian diffusion (FISO), and orientation dispersion index (ODI) maps. More in detail, the NODDI toolbox outputs the isotropic and intraneurite compartments of each voxel, as well as the ODI map, which quantifies angular variation of neurite orientation: the most coherently oriented fibers are the lowest is the ODI value. Then, the output compartments were reparameterized in order to derive the extraneurite compartment, as described in Caverzasi et al. (2016), so that the sum of FICV, FECV, and FISO equaled 1 in each voxel.

NODDI compartment maps were also combined into a single 4D RGB image (red for FECV, green for FICV, and blue for FISO, as in Figure 1a) for visualization and quality‐check purposes, as shown in Caverzasi et al. (2016).

FSL built‐in “dtifit” tool was separately applied to both DTI and HARDI shells (DTI: 35 directions at *b*‐value 711 s/mm^2^; HARDI: 60 directions at *b*‐value 3,000 s/mm^2^) in order to estimate the diffusion tensor, and to generate the following tensorial maps:

- for the DTI shell (Figure 1b): FA, MD, AD, and RD maps;
- for the HARDI shell (Figure 1c): FA, MD, AD, and RD maps.

### 2.5 Tractography

For tractography, HARDI datasets (60 directions; *b*‐value, 3,000 s/mm^2^) were extracted from the NODDI datasets (once preprocessing was completed). Diffusion imaging in Python (Dipy) software (Garyfallidis & Brett, 2014; Soares, Marques, Alves, & Sousa, 2013) was employed to perform tractography that was based on a q‐ball residual bootstrap algorithm (Berman et al., 2008; Caverzasi et al., 2015; Caverzasi, Papinutto, Amirbekian, Berger, & Henry, 2014).

Such algorithm was set as described by Caverzasi et al. (2015) in order to fit the signal to spherical harmonics, to compute the orientation distribution functions, and to identify the primary and principal fiber orientations. Streamline turning angle over a 60° threshold (Caverzasi et al., 2015) and FA below a 0.10 threshold (Bello et al., 2008) were used as stopping criteria; seed density was set at 7^3^ per voxel. Trackvis software (http://trackvis.org) was employed to draw and place single‐plane seed‐ and target‐regions of interest (ROIs), in order to reconstruct the following WM fiber tracts bilaterally (Figure 1d): arcuate fasciculi (AF), frontal aslant tracts (FAT), inferior fronto‐occipital fasciculi (IFOF), uncinate fasciculi (UF), cinguli (CING), corticospinal tracts (CST), and optic radiations (OR). For each fascicle, ROI placement was determined a priori using ROIs either adopted from previous studies or based upon the anatomy of the fascicles as known from other tractography or anatomical studies, as follows. ROIs for AF, IFOF, and UF were placed as in Caverzasi et al. (2015); ROIs for FAT were based on Sanvito et al. (2020); ROIs for CING were based on the fascicle anatomy as known from Wakana, Jiang, Nagae‐Poetscher, van Zijl, and Mori (2004); seed‐ROI and target‐ROI for CST were placed as in Yoo et al. (2019) and Castellano et al. (2012), respectively; ROIs for OR were adapted from Chamberland et al. (2017) and Chamberland, Tax, and Jones (2018) after lateral geniculate nucleus was identified as shown in Kitajima et al. (2015). A more detailed report of anatomical landmarks and references used for ROI placement is displayed in Supplementary Table 1. Once tracking and ROI‐based targeting was completed, for each tract, results were quality‐checked using TrackVis, and all of the following were excluded: obvious artifacts, streamlines directed toward the basal ganglia, toward the contralateral hemisphere, and streamlines representing fascicles other than the one of interest. For each pathological case, an expert neuroradiologist selected the tract of interest as the peritumoral fascicle passing nearest to the tumor that is usually the most clinically relevant for the patient.

### 2.6 Along‐tract stats

Once tractography was performed and tracts were quality‐checked, the MATLAB toolbox *along‐tract‐stats* developed by Colby et al. (http://www.github.com/johncolby/along-tract-stats: Colby et al., 2012) was applied to each subject‐specific tract, in order to obtain along‐tract NODDI‐, HARDI‐ and DTI‐metrics for each tract of each subject (MATLAB2013). Since tractography does not imply any directional information, raw streamlines were manually reoriented according to a common origin, corresponding to the cranialmost portion of the tract for FAT; the frontalmost portion of the tract for AF, IFOF, UF, CING, and OR; the caudalmost portion of CST. Fiber origin was selected by an expert neuroradiologist, both for healthy and pathological cases. A lookup table of the 100 points selected along each tract shows how the extremities were not considered by the algorithm, to guarantee better intrasubject reproducibility and focus on the most consistent portion of fiber tracts (Supplementary Figure 1). Streamlines were reparameterized with cubic B‐splines and automatically resampled by the algorithm into 100 points (vertices) evenly distributed along their lengths, as already recommended by Colby et al. (2012). Thus, the n‐vertex of each streamline could easily correspond to the *set* of n‐vertices belonging to other streamlines within the same tract (Figure 1e). The toolbox also generated a “skeleton tract” obtained by averaging the spatial coordinates of all streamline vertices within one fiber tract (i.e., by “collapsing” the vertices, Figure 1f) that can be useful for a synoptic inspection of the resampling. Each subject‐specific NODDI/HARDI/DTI map was then resampled, as well, in order for the voxels to match the vertices of the streamlines, and NODDI/HARDI/DTI metrics were extracted from the resampled voxels. For each *set* of vertices along the tract, the toolbox outputs included: (a) a mean scalar value representing the cross‐sectional mean of each NODDI/HARDI/DTI metric along the tract and (b) the corresponding *SD*. Plotting the cross‐sectional mean of each metric allowed to visualize the profile of the metric of interest along each tract, both in healthy controls and in patients (Figure 1g).

### 2.7 Healthy controls' cohort: Internal reference database of microstructural profiles of healthy fiber tracts and statistical analyses

Microstructural metrics along each healthy subject's fiber tracts were gathered in an internal reference database. From each tract‐specific cross‐sectional means of the NODDI/HARDI/DTI metrics, we discarded the values corresponding to the points number 1, 2, 99, and 100. This operation was considered necessary after realizing that cross‐sectional means assumed outlying values in the proximity of the streamline endpoints. For each metric along each fiber tract, the remaining 96 cross‐sectional mean values obtained from all the healthy subjects were plotted together in order to obtain metric‐specific “healthy microstructural profiles” of each fiber tract, showing the mean, *SD*, and 95% confidence intervals of the cross‐sectional means from all the 15 healthy controls.

In order to assess the consistency of metrics across the 15 subjects, the number of outlying values was computed from each metric of every tract, through two independent statistical tests. Values were considered outliers both according to the ROUT test (setting Q = 1% as the maximum desired false discovery rate) and when higher than Q3 + 1.5*IQR, or lower than Q1–1.5*IQR (IQR being the point‐specific interquartile range). Then, to describe the variability of our internal reference database, the coefficient of variation (CV) of all corresponding points was computed (% of *SD*/mean), and its median and quartiles were obtained for each metric. Supplementary Table 2 summarizes the number of outliers and the CV distribution across the dataset. Further descriptive statistics (i.e., minimum and maximum *SD*, minimum and maximum confidence interval width) of the cross‐sectional means of NODDI/HARDI/DTI metrics along each fiber tract were summarized in Table 2 and Supplementary Table 3 (see Section 3).

**TABLE 2:** Descriptive statistics of the normative reference NODDI‐derived metrics from the healthy controls. For each of the 96 points along the tracts, a cross‐sectional mean from each subject was extracted, and *SD* and 95% CI width of the means from all subjects were calculated. This table reports minimum and maximum values of *SD* and 95% CI width

	Normative NODDI metrics	Left side				Right side			
		Min *SD*	Max *SD*	Min 95% CI width	Max 95% CI width	Min *SD*	Max *SD*	Min 95% CI width	Max 95% CI width
Arcuate fascicle	FICV	0.0177	0.0340	0.0196	0.0376	0.0162	0.0407	0.0178	0.0452
	FECV	0.0235	0.0356	0.0262	0.0394	0.0238	0.0536	0.0264	0.0594
	FISO	0.0124	0.0279	0.0137	0.0308	0.0122	0.0270	0.0135	0.0300
	ODI	0.0154	0.0420	0.0170	0.0466	0.0194	0.0400	0.0214	0.0442
Frontal aslant tract	FICV	0.0227	0.0402	0.0252	0.0444	0.0215	0.0407	0.0238	0.0452
	FECV	0.0277	0.0801	0.0306	0.0888	0.0231	0.0737	0.0256	0.0816
	FISO	0.0104	0.0986	0.0116	0.1092	0.0141	0.0837	0.0156	0.0926
	ODI	0.0212	0.0882	0.0234	0.0978	0.0163	0.0810	0.0180	0.0896
IFOF	FICV	0.0198	0.0351	0.0220	0.0388	0.0177	0.0437	0.0196	0.0484
	FECV	0.0278	0.0590	0.0308	0.0654	0.0244	0.0439	0.0270	0.0486
	FISO	0.0193	0.0650	0.0213	0.0720	0.0156	0.0606	0.0173	0.0672
	ODI	0.0143	0.0427	0.0158	0.0474	0.0083	0.0375	0.0092	0.0416
Uncinate fascicle	FICV	0.0199	0.0526	0.0220	0.0582	0.0208	0.0570	0.0230	0.0632
	FECV	0.0337	0.0873	0.0372	0.0966	0.0289	0.0692	0.0320	0.0766
	FISO	0.0281	0.0790	0.0312	0.0876	0.0222	0.0612	0.0246	0.0678
	ODI	0.0166	0.0453	0.0184	0.0500	0.0272	0.0632	0.0302	0.0702
Cingulum	FICV	0.0264	0.0618	0.0292	0.0686	0.0309	0.0449	0.0342	0.0498
	FECV	0.0263	0.0601	0.0290	0.0666	0.0396	0.0617	0.0438	0.0682
	FISO	0.0136	0.0395	0.0150	0.0437	0.0196	0.0394	0.0217	0.0436
	ODI	0.0215	0.0744	0.0238	0.0824	0.0265	0.0559	0.0294	0.0618
Corticospinal tract	FICV	0.0197	0.0932	0.0218	0.1032	0.0190	0.1147	0.0210	0.1270
	FECV	0.0204	0.0965	0.0226	0.1068	0.0201	0.0892	0.0222	0.0988
	FISO	0.0102	0.1452	0.0112	0.1608	0.0092	0.1805	0.0102	0.2000
	ODI	0.0137	0.0498	0.0152	0.0552	0.0131	0.0493	0.0144	0.0546
Optic radiation	FICV	0.0253	0.0825	0.0280	0.0912	0.0269	0.0520	0.0298	0.0576
	FECV	0.0290	0.0820	0.0322	0.0908	0.0273	0.0619	0.0302	0.0686
	FISO	0.0211	0.1105	0.0233	0.1224	0.0168	0.0789	0.0186	0.0872
	ODI	0.0195	0.0393	0.0216	0.0434	0.0183	0.0444	0.0202	0.0492
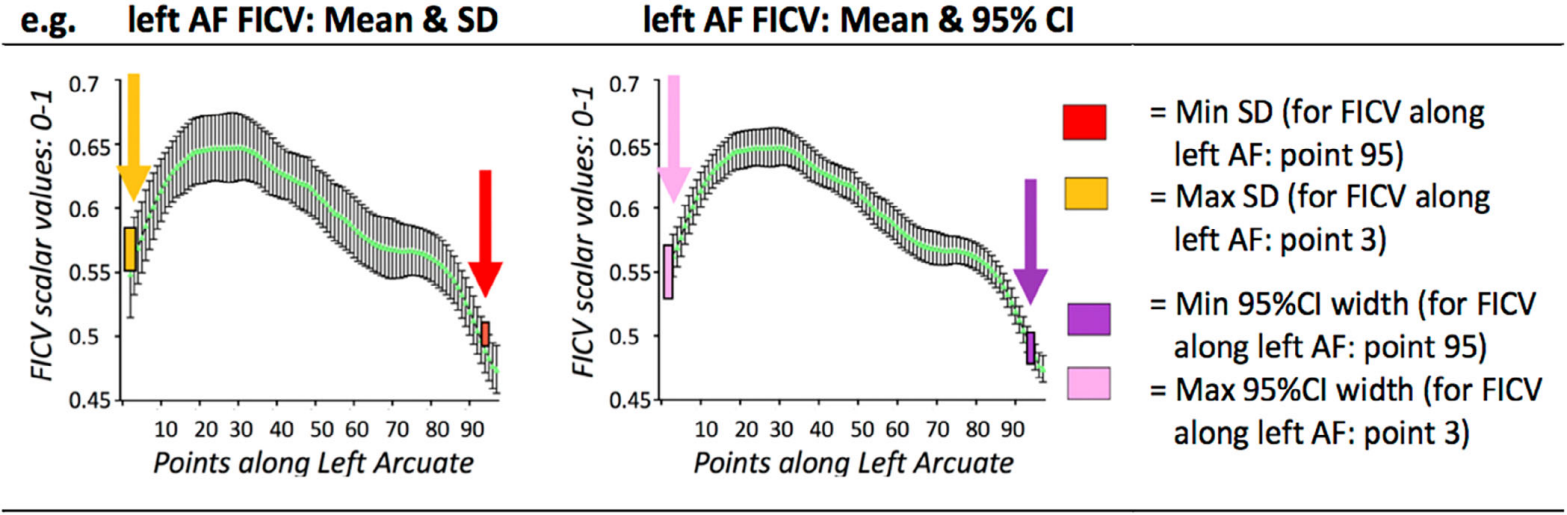									

Abbreviations: FECV, fraction of extracellular volume; FICV, fraction of intracellular volume; FISO, fraction of intracellular volume; NODD, neurite orientation dispersion and density imaging; ODI, orientation dispersion index.

In addition, to evaluate the relationship between NODDI and HARDI/DTI metrics, Spearman's rank correlation coefficient (*r*) was calculated between FICV and FECV, FA and ODI, and FA and FICV.

Finally, in order to assess whether each fiber tract showed hemispheric asymmetry in the diffusion metrics, each value along each fiber tract was compared to its contralateral counterparts, as follows. For each metric extracted from each point along the tract, the cross‐sectional means from the healthy controls were compared to the contralateral cross‐sectional means (e.g., along‐tract FICV of Point 3 of left arcuate fasciculus vs. along‐tract FICV of Point 3 of right arcuate fasciculus, etc.) using Wilcoxon matched pairs signed rank tests, applied to each one of the 96 points. A nonparametric test was picked because not all of the cross‐sectional mean sets showed a Gaussian distribution: some of the sets failed a D'Agostino and Pearson normality test. After Wilcoxon test, a Bonferroni correction was applied, taking in consideration that 96 comparisons were performed for each tract‐specific diffusion metric. We considered the metrics significantly asymmetric exclusively for the tract points for which Wilcoxon test showed a *p* < .05 after Bonferroni correction.

### 2.8 Patients' cohort: Comparing patient‐specific fiber tract profile to the microstructural profiles of healthy fiber tracts

Since brain neoplasms cause differently distributed microstructural abnormalities, depending on the tumor site, affecting different portions of different fiber tracts, patients' data were analyzed at the single‐subject level. Tractography fascicle models and DTI/NODDI maps were coregistered to FLAIR and postcontrast T1 images for visualization, quality‐check, and evaluation of the relationship between tracts and tumor. Fiber tracts whose trajectories were adjacent to the tumor location were selected, and, for each NODDI/HARDI/DTI metric, the microstructural profiles obtained from the central 96 cross‐sectional mean values were compared to the corresponding microstructural profiles of healthy fiber tracts and plotted in line graphs. First, a qualitative comparison was performed by visually evaluating the plots, aiming at unraveling how different metrics changed with respect to each other in pathological conditions, and whether NODDI‐derived metrics accounted for the pathological microstructural changes of the peritumoral tissue more accurately than DTI‐derived ones. Then, a quantitative comparison was performed by calculating the extent to which the patient's metrics diverged from the controls' reference database of healthy microstructural profiles, aiming at evaluating whether this divergence is present both in the peritumoral tract and in the contralateral one. The divergence of the patient‐specific profile from the healthy microstructural profile was considered significant for every point of the profile in which the patient's metric exceeded ±≥2 *SD*s from the mean of the healthy controls.

## 3 RESULTS

### 3.1 Healthy controls' cohort: “Healthy microstructural profiles” of diffusion metrics

In the healthy controls' cohort, dMRI‐derived diffusion metrics were extracted along the trajectory of all the seven reconstructed WM bundles, bilaterally, and are organized in an internal reference dataset. Results are visually reproducible among the 15 subjects and, statistically, a very low percentage of observations is identified as outliers (0.35% of observations on average, ROUT test; 1.71% on average, IQR test—refer to Supplementary Table 2 for details). DTI‐derived (*b* = 711 s/mm^2^) AD quantified along the fibers always displays higher absolute values with respect to MD and RD, and its course is specular to RD in all tracts. MD curves lay in the middle, as expected (Supplementary Figure 2a). Notably, all HARDI‐derived metrics show patterns analogous to the DTI‐derived ones, but lower absolute values (Supplementary Figure 2b). Further descriptive statistics of DTI‐ and HARDI‐derived diffusion metrics extracted from 96 points (cross‐sectional means) along each tract in the population of healthy controls are shown in Supplementary Table 2.

**FIGURE 2:**
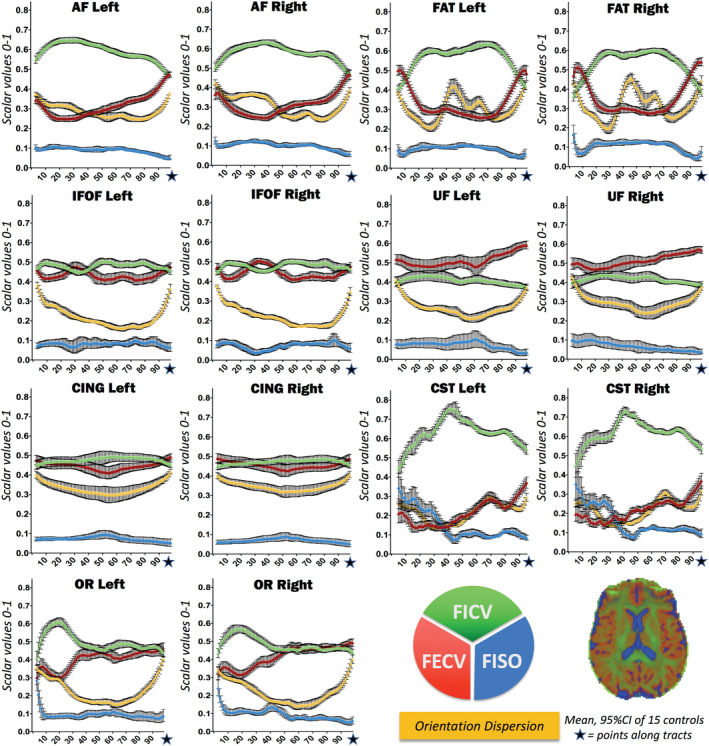
Normative reference of neurite orientation dispersion and density imaging (NODDI) metrics along all tracts: fraction of intracellular volume (FICV), fraction of extracellular volume (FECV), fraction of intracellular volume (FISO), and orientation dispersion index (ODI). Mean and 95% CI of the cross‐sectional means from 15 healthy controls computed for each NODDI‐derived metric are displayed in line graphs: FICV (green), FECV (red), FISO (light blue), and ODI (yellow). Supplementary Figure 3 shows how FICV and FECV are negatively correlated

**FIGURE 3:**
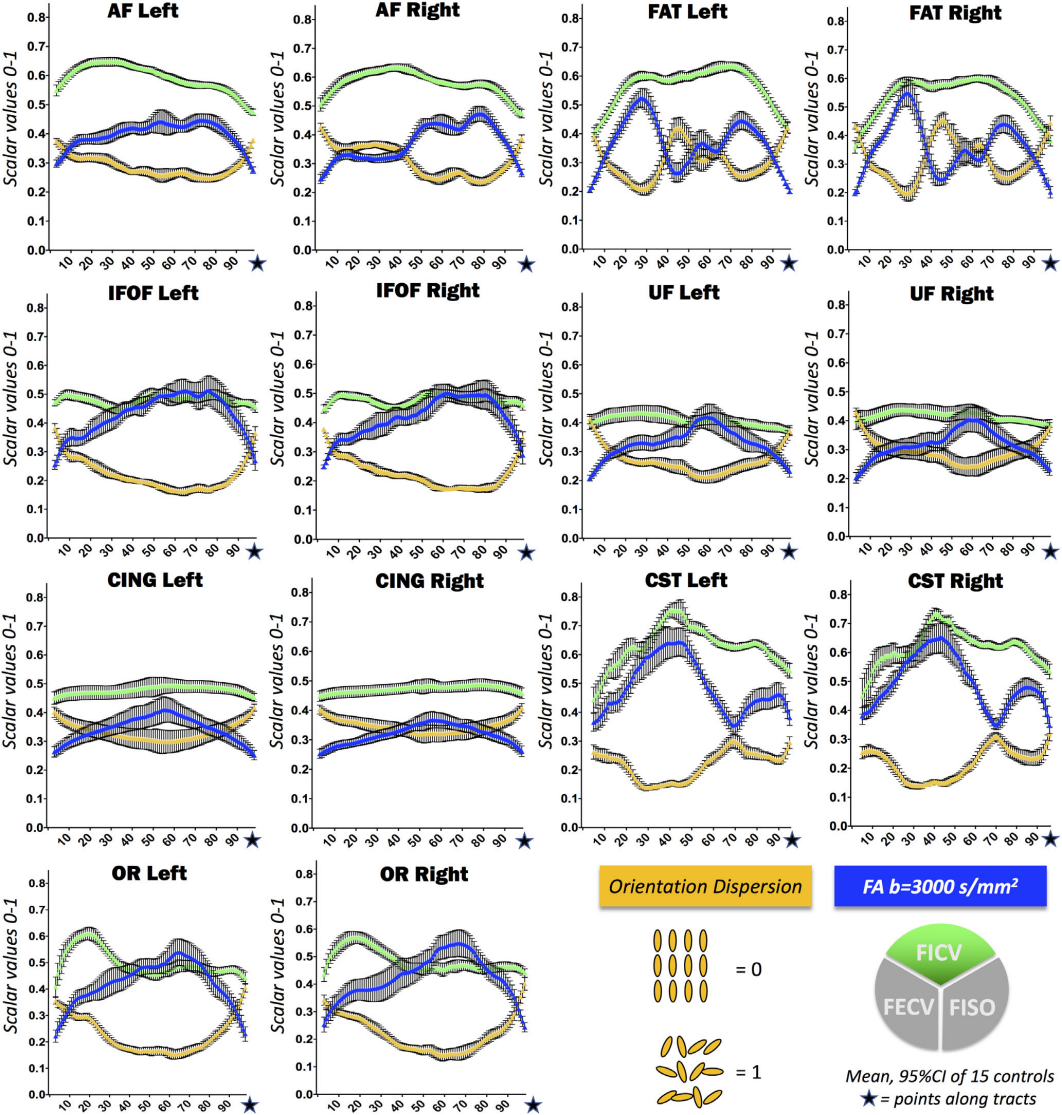
Normative reference of neurite orientation dispersion and density imaging (NODDI) metrics along all tracts: fraction of intracellular volume (FICV), fractional anisotropy (FA), and orientation dispersion index (ODI). Mean and 95% CI of the cross‐sectional means from 15 healthy controls computed for FICV (green), fractional anisotropy (FA) (blue), and ODI (yellow) are shown together in order to highlight the reciprocal relationships between the curves. In all tracts, FA is inversely correlated to the ODI profile, and only partially influenced by FICV values. Table 1 provides further statistics regarding the cross‐sectional means, and Supplementary Figures 4 and 5 show details on metric correlations

The computation of along‐tract NODDI‐derived metrics reliably segregates signals arising from the intraneurite (FICV), extraneurite (FECV), and free water (FISO) compartments for the entire trajectory of the fibers. Along all healthy fascicles, free water is almost absent, and the highest intracellular volume (FICV) is mirrored by the lowest FECV (Figure 2), with a significant inverse correlation in all fascicles (Supplementary Figure 3). In addition, our analysis allows to disentangle the two principal FA determinants: ODI and FICV (Figure 3). In all seven WM fascicles, FA profiles maintain a strong inverse correlation to ODI (Spearman's *R* < −.96 and *p*‐value <.0001 for each fascicle). On the contrary, a weaker positive correlation is found between FA and FICV. Statistical correlations between metrics are shown in Supplementary Figures 4 and 5; descriptive statistics of novel NODDI‐derived diffusion metrics quantified along healthy WM fiber bundles are displayed in Supplementary Table 2 and Table 2.

**FIGURE 4:**
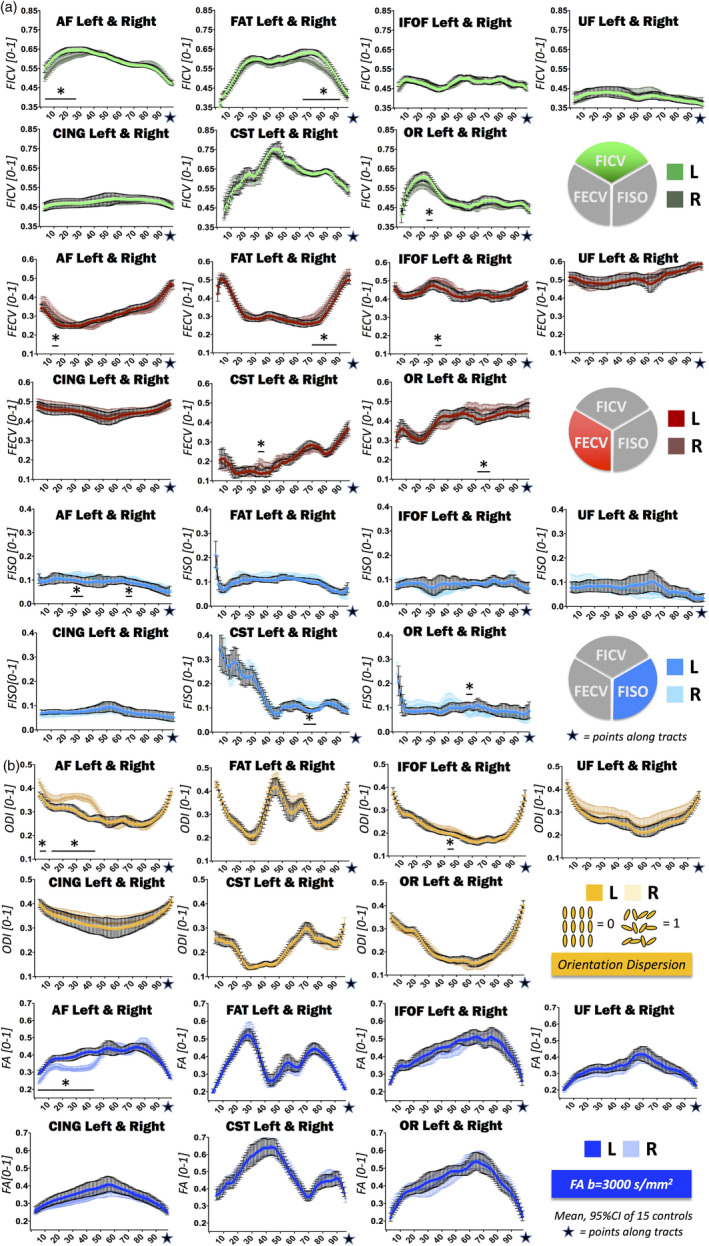
Comparison of neurite orientation dispersion and density imaging (NODDI) metrics between left and right hemispheres. Wilcoxon matched pairs signed rank tests and subsequent Bonferroni corrections were applied in order to disclose differences in along‐tract NODDI metrics between the right and left hemispheres. Significant differences are highlighted in line graphs (* = *p* < .05 after Bonferroni correction). (a) Fraction of intracellular volume (FICV) (green), fraction of extracellular volume (FECV) (red), fraction of intracellular volume (FISO) (light‐blue) and (b) orientation dispersion index (ODI) (yellow), and fractional anisotropy (FA) (blue)

Finally, subtle hemispheric asymmetries have been highlighted by the paired comparison applied to individual diffusion metrics of each healthy tract. In particular, significant asymmetries between the left and right brain side emerge in the FICV of AF (higher on the left: points [5–24], [27]), FAT (higher on the left: points [68–90]), and OR (higher on the left: points [21–24]), in the FECV of AF (higher on the right: points [11–13]), FAT (higher on the right: points [72–85], [89], [92], [95]), IFOF (higher on the right: points [34–35]), CST (higher on the right: points [32–34]), and OR (higher on the right: points [66–69]), in the free fluid of AF (higher on the right: points [27–35]; [69–70]), CST (higher on the right: points [66–72]), and OR (higher on the left: points [54–56]) (Figure 4a), in the orientation dispersion of AF (higher on the right: points [3–13]; [18–43]) and IFOF (higher on the right: points [47–48]), and in the FA of AF (higher on the left: points [4–43]) (Figure 4b).

AF is the only tract with consistent hemispheric asymmetries in corresponding points across both DTI‐ and NODDI‐metrics, with the left AF presenting higher FICV, higher FA, and lower FECV and ODI with respect to its right counterpart. In fact, FICV–FECV–ODI–FA asymmetries overlap in points [11–13]; FICV–ODI–FA asymmetries overlap in points [5–13], [18–24], [27]; FISO‐ODI‐FA asymmetries overlap in Points 27–35 (Figure 4a,b). While AF asymmetry is clearly highlighted also by the DTI‐metric analysis, FAT asymmetry is exclusively illustrated by the NODDI‐metric analysis. Indeed, left FAT shows significantly higher FICV for a considerable portion of the tract (points [68–90]), associated with a lower FECV (points [72–85], [89], [92], [95]) (Figure 4a).

### 3.2 Patients' cohort: Detecting alterations along peritumoral WM tracts

In the patients' cohort, WM tracts were consistently reconstructed in all cases. Eloquent peritumoral fiber bundles were selected for qualitative assessment and quantification of dMRI‐derived diffusion metrics. No significant differences with the healthy microstructural profiles are found in tracts contralateral to the tumors. Comparing pathological microstructural profiles of peritumoral fascicles to the “healthy microstructural profiles” enables to identify some recurrent patterns across patients. Such patterns describe how FA (HARDI‐derived, *b* = 3,000 s/mm^2^) and NODDI‐metrics mutually diverge from the reference profiles, as illustrated in the following paragraphs with the aid of representative case figures and plots. As an additional analysis, along‐tract MD (both at *b* = 711 s/mm^2^ and *b* = 3,000 s/mm^2^) was evaluated and compared to NODDI‐metrics.

#### 3.2.1 Pattern A: Decreased FICV, increased FECV, normal FA

*Pattern A* is the most commonly observed pattern (12 out of 22 subjects, 54.5%, Supplementary Figure 6a) and consists in a FICV decrease associated with a FECV increase. Both metrics significantly diverge from the reference profiles in the same points where FA is not significantly affected, or is affected in a remarkably shorter segment of the tract.

In 9 cases out of 12 (Patients #3–#11 in Supplementary Figure 6a; 75% of subjects presenting with *Pattern A*), FA is not significantly affected at any along‐tract points of the peritumoral fiber tracts, whereas along‐tract FICV and FECV show significant divergence from the healthy profiles.

In these cases, altered NODDI metrics represent the only noninvasive evidence of peritumoral WM suffering and microstructural modifications. Furthermore, along‐tract FICV seems to be more sensitive than along‐tract FECV, reflecting a microstructural abnormality of a longer segment of the fascicle.

In the remaining 3 cases out of 12 (Patients #1, #2, and #12 in Supplementary Figure 6a; 25%), also along‐tract FA is significantly reduced in some points, but FICV and FECV show a significant alteration for a remarkably longer segment of the tract.

**FIGURE 5:**
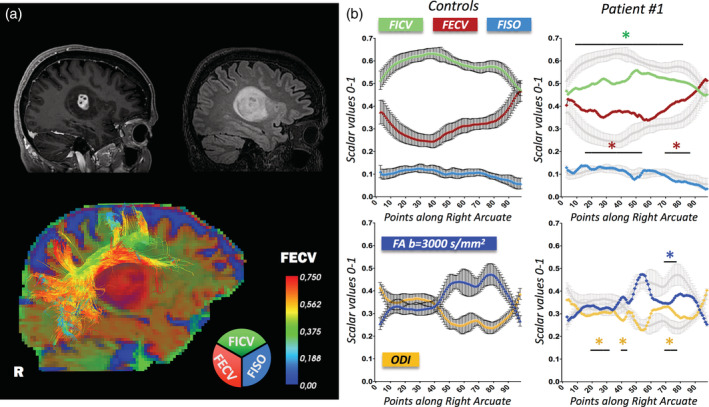
Peritumoral arcuate fasciculus (AF) in glioblastoma. Along‐tract diffusion metrics of a right AF in the proximity of a glioblastoma (WHO IV). (a) 3DT1 preoperative imaging is shown in the top‐left corner, 3DFLAIR in the top‐right corner, neurite orientation dispersion and density imaging (NODDI)‐compartment RGB map in the bottom image. R = right side. (b) Mean ± *SD* of reference metrics derived from the 15 healthy controls are displayed on the left (color‐coded). Patient's metrics are displayed on the right (color‐coded), overlaid on the mean ± *SD* of reference metrics (gray). Divergences of more than ±2 *SD* from “healthy microstructural profiles” are reported. Fraction of intracellular volume (FICV): decreased in points [5–86]; fraction of extracellular volume (FECV): increased in points [15–52], [67–86]; fraction of intracellular volume (FISO): no divergences; orientation dispersion index (ODI): decreased in points [18–33], [36–41], increased in points [68–78]; fractional anisotropy (FA): decreased in points [68–76]. Along‐tract mean diffusivity (MD), extracted both at *b* = 711 s/mm^2^ and *b* = 3,000 s/mm^2^, is displayed in Supplementary Figure 7a

As a representative case for *Pattern A*, we selected Patient #1 (Figure 5), a 60‐year‐old man with a right temporo‐insular glioblastoma, presenting with recurrent left motor epileptic seizures and mild dysarthria. Preoperative HARDI tractography shows an intact right AF, but our quantitative analysis reveals microstructural alterations. NODDI‐derived diffusion metrics significantly deviate from the reference curves in many different points. The major alteration can be identified in the frontal portion of the tract that is in close proximity to the tumor. In fact, patient's FICV is lower than the “healthy microstructural profiles” in points [5–86], FECV is higher in points [15–52], [67–86], ODI is lower in points [18–33], [36–41] and higher in points [68–78]. Conversely, patient's FA fails to disclose most of those tissue‐specific modifications, resulting within 2 *SD* from the normal range until Point 67 of the tract. Pathological FA significantly diverges from the internal reference standard only in points [68–76] that correspond to the AF peritrigonal portion.

#### 3.2.2 Pattern B: Decreased FICV, increased FECV, paradoxically increased FA

*Pattern B* is observed where the peritumoral tract segments show FICV decrease and FECV increase in the peritumoral tract segments, associated with a paradoxical FA increase (8 out of 22 subjects, 36.4%, Supplementary Figure 6b).

In 3 cases out of 8 (Patients #13, #19, and #20 in Supplementary Figure 6b, 37.5% of patients with *Pattern B*), the FA increase is observed in tract portions also characterized by along‐tract FICV and FECV anomalies. In one case out of eight (Patient #14 in Supplementary Figure 6b, 12.5%), the FA increase partially colocalizes with the along‐tract FICV and FECV anomalies. In the remaining four cases out of eight (Patients #15–#18 in Supplementary Figure 6b, 50%) the paradoxical FA increase is observed in tract portions where along‐tract NODDI metrics are not altered, whereas FA is in range in the points corresponding to FICV decrease and FECV increase.

In all these cases, FA increase perfectly colocalizes with ODI decrease. This paradoxical finding may be explained by the strong influence of ODI (significantly reduced in the corresponding points) on FA, as commented in Section 4.

**FIGURE 6:**
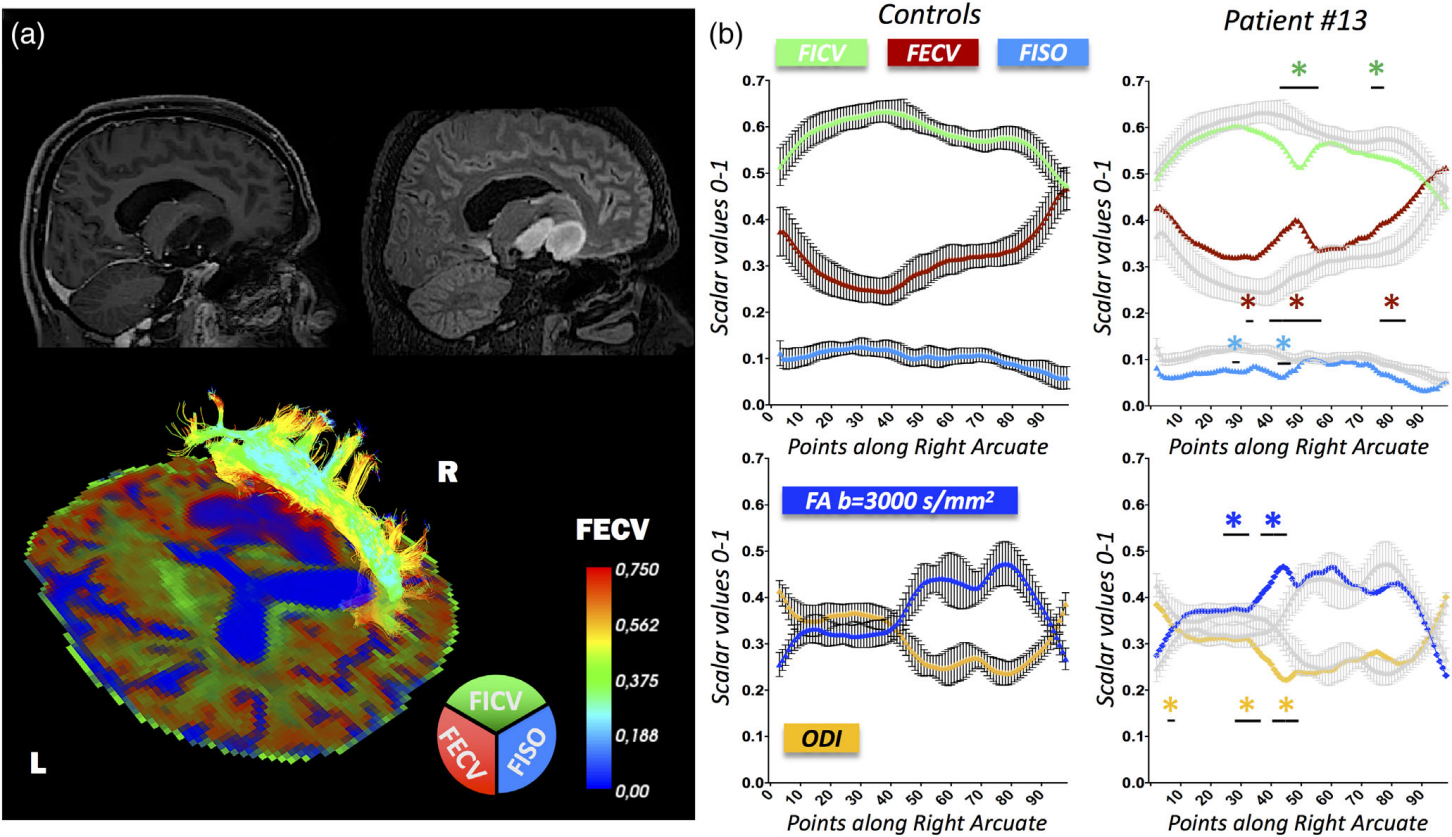
Peritumoral arcuate fasciculus (AF) in astrocytoma grade III. Along‐tract diffusion metrics of a right AF in the proximity of an astrocytoma (WHO III). (a) 3DT1 preoperative imaging is shown in the top‐left corner, 3DFLAIR in the top‐right corner, neurite orientation dispersion and density imaging (NODDI)‐compartment RGB map in the bottom image. R = right side; L = left side. (b) Mean ± *SD* of reference metrics derived from the 15 healthy controls are displayed on the left (color‐coded). Patient's metrics are displayed on the right (color‐coded), overlaid on the mean ± *SD* of reference metrics (gray). Divergences of more than ±2 *SD* from “healthy microstructural profiles” are reported. Fraction of intracellular volume (FICV): decreased in points [44–57], [74–76]; fraction of extracellular volume (FECV): increased in Point 33, [37–55], [75–85]; fraction of intracellular volume (FISO): decreased in Point 33, [44–48]; orientation dispersion index (ODI): decreased in Point 3, points [24–32], [36–47]; fractional anisotropy (FA): increased in points [22–32], [36–47]. Along‐tract mean diffusivity (MD), extracted both at *b* = 711 s/mm^2^ and *b* = 3,000 s/mm^2^, is displayed in Supplementary Figure 7b

As a representative case for *Pattern B*, we selected Patient #13 (Figure 6), a 30‐year‐old patient diagnosed with insular and capsular disease progression of a relapsing astrocytoma grade III in the right temporal lobe. Presurgical HARDI imaging allowed the right AF reconstruction; along‐tract NODDI analysis showed that right AF FICV diverges from “healthy microstructural profiles” in points [44–57], [74–76], and its FECV deviates in points [33], [37–55], [75–85]. In the exact portion of the right AF corresponding to the lowest FICV (points [44–47]), the FA profile shows an unexpected significant increase with respect to the reference values, extended also to points [22–32], [36–47].

#### 3.2.3 Patterns C and D

The remaining two patients did not fall into the abovementioned patterns, and showed peculiar abnormalities of diffusivity metrics, that we named Patterns C and D for consistency, respectively.

**FIGURE 7:**
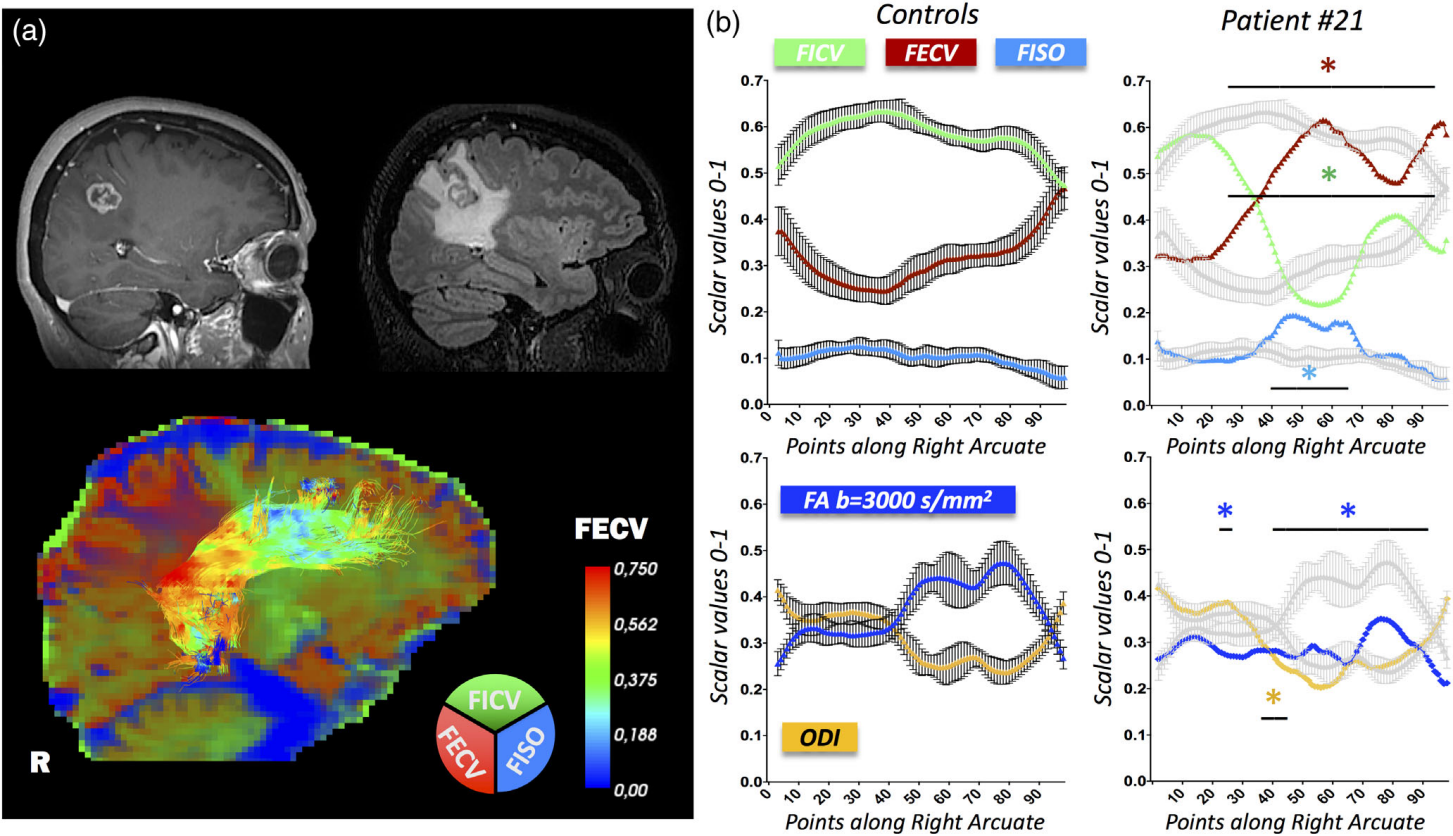
Peritumoral arcuate fasciculus (AF) in brain metastasis. Along‐tract diffusion metrics of a right AF in the proximity of a brain metastasis from lung cancer. (a) 3DT1 preoperative imaging is shown in the top‐left corner, 3DFLAIR in the top‐right corner, neurite orientation dispersion and density imaging (NODDI)‐compartment RGB map in the bottom image. R = right side. (b) Mean ± *SD* of reference metrics derived from the 15 healthy controls are displayed on the left (color‐coded). Patient's metrics are displayed on the right (color‐coded), overlaid on the mean ± *SD* of reference metrics (gray). Divergences of more than ±2 *SD* from “healthy microstructural profiles” are reported. Fraction of intracellular volume (FICV): decreased in points [24–98]; fraction of extracellular volume (FECV): increased in points [23–98]; fraction of intracellular volume (FISO): increased in points [42–69]; orientation dispersion index (ODI): decreased in points [37–45]; fractional anisotropy (FA): decreased in points [24–27], [41–98]. Along‐tract mean diffusivity (MD), extracted both at *b* = 711 s/mm^2^ and *b* = 3,000 s/mm^2^, is displayed in Supplementary Figure 7c

Patient #21 (*Patten C*, Supplementary Figure 6c and representative case in Figure 7) was a 49‐year‐old patient presenting with sudden confusion and tonic–clonic seizures, due to a right parietal metastatic brain tumor. Diffusion‐derived quantitative metrics along her right AF emphasize severe WM microstructural damage, with all the metrics resulting significantly abnormal with respect to the “healthy microstructural profiles.” Significantly decreased values can be appreciated for FICV (points [24–98]), ODI (points [37–45]), and FA (points [24–27], [41–98]) along a conspicuous portion of the tract profile. A significantly increased FECV is measured from points [23–98], while a circumscribed FISO upsurge is evident along points [42–69]. In this case, both DTI‐ and NODDI‐metrics revealed severe WM alterations, but NODDI‐metrics provided additional insight regarding the reciprocal modifications of water compartments.

Finally, the case of patient #22 (*Patten D*, Supplementary Figure 6d) was characterized solely by an FISO along‐tract upsurge, in the absence of any other abnormal values of the remaining along‐tract diffusivity metrics.

#### 3.2.4 Comparing NODDI‐metrics and MD

An additional analysis (Supplementary Figure 7) was performed on MD. Along‐tract MD extracted from different shells (*b* = 711 s/mm^2^ and *b* = 3,000 s/mm^2^) exhibited the same profile shape, but different values (higher at lower *b*‐value). Unlike FA variously displaying increased or decreased values, MD from both shells in the peritumoral tracts was either in range or increased, therefore being supposedly related to anomalies in the extracellular compartment. NODDI‐metrics were overall more sensitive than MD for detecting WM peritumoral alterations. Indeed, approximately half of the patients (12 out of 22, 54.5%) exhibited more extensive or more pronounced alterations in NODDI‐metrics than in MD, and for 6 of these patients (#6, #8, #12, #16, #17, #22) the increased sensitivity of NODDI‐metrics was particularly evident (Supplementary Figure 7).

## 4 DISCUSSION

In this study, diffusion metrics derived from the NODDI analysis have been integrated with the along‐tract statistics tool proposed by Colby et al. (2012), and thoroughly quantified along seven different WM fiber tracts, bilaterally, in 15 healthy subjects and 22 patients with brain tumors. We built an internal standard reference database containing NODDI‐ and DTI‐metrics along WM tracts of healthy controls and we used it to plot tract‐specific metric‐specific healthy microstructural profiles that can be compared with patient cases. The main finding of the study is that along‐tract NODDI metrics are more sensitive in detecting anatomical asymmetries in the healthy brain and, also, more sensitive than DTI‐metrics in identifying subtle tract‐specific peritumoral microstructural changes.

Recently, along‐tract computation of DTI‐derived diffusion metrics has been achieved by means of different algorithms. O'Donnell et al. implemented a tract‐based morphometry method to detect subtle hemispheric asymmetry of MD and FA along the CING and AF of healthy subjects, to highlight that those quantitative measures spatially vary along tract trajectories and that the mean of a scalar value in the entire tract may be inadequate to describe minimal WM changes (O'Donnell et al., 2009). Furthermore, with the aim of facilitating data sharing and dissemination of this powerful type of tractometry analysis despite its computational complexity, Colby et al. and Yeatman et al. provided two different publicly available tools to conduct along‐tract measurements, potentially customizable by future users depending on their exigencies. Above all, Colby et al. demonstrated their tool extensibility by performing between‐group analyses and comparing the FA along the inferior longitudinal fascicle and AF of children with fetal alcohol spectrum disorders and controls (Colby et al., 2012). Additionally, a step forward has been taken by Yeatman et al. by sharing a normative distribution of DTI‐derived MD, RD, and FA along different WM bundles in healthy brains, so that individual's tract profiles of patients with multiple sclerosis (MS) could be compared to the healthy tract profiles in a plot (Yeatman et al., 2018). Further studies performing along‐tract analysis of DTI metrics were published by Talozzi et al. (2018) and Chen, Zhang, Yushkevich, Liu, and Beaulieu (2016).

Being interested in analyzing pathological metrics extracted from peritumoral WM fibers, we aimed at integrating DTI‐metrics with the advanced NODDI‐metrics that can be more accurate in unraveling restrained microstructural tissue derangements. Thus, we implemented a pipeline to extract both DTI‐ and NODDI‐derived quantitative diffusion metrics along WM tracts, and we applied it both to healthy subjects and patients with brain tumors.

### 4.1 Healthy controls' cohort

The reproducibility and reliability of our working pipeline was assessed by extracting along healthy fascicles classical DTI metrics such as FA, AD, MD, and RD, and confirming that their profiles follow the same pattern as the corresponding DTI‐curves previously published. Our along‐tract DTI metrics analyses appeared consistent and reliable, capable of depicting the waved profile of diffusion metrics instead of averaging all voxels of WM bundles; we thus moved to measure NODDI metrics along all healthy tracts. The added value of NODDI parameters over DTI‐derived metrics is known to rely on a more regionally specific characterization of tissue microstructure, by revealing WM composition more reliably than FA or MD (Reddy & Rathi, 2016; Timmers et al., 2016). A normative dataset of NODDI inherently microscopic measures along healthy fiber tracts is a powerful tool to establish a normative range of values where WM can be considered as nonpathological. In this work, for the first time, we provide plots representing the signature “healthy microstructural profiles” of NODDI metrics extracted along the principal WM fiber tracts, together with tables that summarize the corresponding descriptive statistics.

Moreover, the point‐by‐point quantification of the contribution of distinct diffusion compartments to the total diffusion signal allows to appreciate hemispheric asymmetries.

AF shows significant asymmetry of both DTI‐ and NODDI‐metrics, more remarkable in the frontal portion of the tract. In particular, left AF shows higher FA, higher FICV, and lower orientation dispersion than right AF. Conversely, FAT shows significant asymmetry exclusively of NODDI‐metrics (FICV higher on the left, in particular), whereas the DTI‐analysis does not highlight any significant asymmetry. Interestingly, such asymmetry is specific for the caudalmost portion of the tract (i.e., the inferior frontal branch). This result advocates for a higher sensitivity of NODDI‐metrics that are capable of detecting more subtle microstructural variants when compared to DTI‐metrics. These observations regarding the asymmetry of FAT and AF are consistent with them having a major role in the dorsal phonological‐articulatory stream of language (Friederici, 2012; Hickok & Poeppel, 2007) that is known to be strongly left‐hemisphere dominant (Hickok & Poeppel, 2007). Indeed, such metric asymmetry can be interpreted as a higher FICV and fiber coherence of these dorsal stream language tracts in the left‐hemisphere. On the other hand, metric asymmetry displayed by the other tracts are evident only for few metrics and for very limited tract portions, consistently with the notion that the neural networks they belong to motor system, visual system, and ventral semantic system of language are less hemisphere specific.

Finally, we further demonstrated that FA values in the WM, and more specifically along tracts, strongly depends on ODI, whereas is influenced by FICV (and FECV) more weakly. This finding is consistent with previous studies (Zhang et al., 2012) and suggests that FICV and FECV variations could reflect WM abnormalities in pathological conditions without necessarily co‐localizing with FA alterations. Besides providing information about healthy microstructural profiles and their asymmetry, this normative database served as an intrastudy reference for comparison to pathological cases, representing an example of a new method for the detection of previously indiscernible WM modifications in a broad spectrum of neurological and psychiatric diseases in adult subjects.

### 4.2 Patients' cohort

Since group‐analysis in patients was not possible due to differently distributed tissue abnormalities, we compared data extracted from patients with brain tumors, at the single‐subject level, to the healthy cohort. First, the divergence between NODDI curves of pathological metrics and their corresponding reference standard was clearly evident, both at a qualitative visual assessment and after point‐by‐point statistical analyses. Relevantly, changes in the microstructural metrics are always confined to a specific peritumoral WM area. Thus, these data remark that the quantitative assessment of diffusion‐derived metrics in the peritumoral WM is pivotal to more specifically define tumor extension, infiltration, and biological behavior (Castellano & Falini, 2016). Since actual tumor margins can extend far beyond the ones detected by conventional MRI, tumor dimensions may be underestimated during presurgical planning, leading to incomplete resection and subsequent worse prognosis for the patient. Convincing evidence showed that changes in classical DTI metrics can pinpoint minor WM derangements caused by occult cancer infiltration, although the correlation between those alterations and tissue microstructural properties is flawed, since DTI‐derived quantitative parameters are influenced by a multiplicity of tissue‐specific biological properties (Cortez‐Conradis et al., 2013; Sternberg, Lipton, & Burns, 2014). The more accurate along‐tract spatial definition of WM alterations, the more efficient patient‐tailored management can be achieved. In addition, whereas NODDI‐analysis itself can provide information about peritumoral WM, along‐tract NODDI‐analyses can add relevant pieces of information regarding which WM fiber tract is affected by the microstructural abnormalities, and to what extent (compared to the healthy controls' reference database).

Another important point emerging from our results regards the interpretation of FA along pathological tracts, sometimes aspecific or even misleading.

Despite being the diffusion metric most commonly studied to define microstructural tissue alterations, in fact, FA may not specifically reflect real biological processes (Alexander et al., 2017). In particular, decreased FA values may reflect reduced myelination, reduced axonal density, increased neurite dispersion, or even just a DTI methodological failure in correctly quantifying the crossing fibers. The macroscopic tissue anisotropy described by FA has two main contributing factors at the microscopic level, that could be disentangled by the NODDI analysis, but not by the classical DTI: *microscopic anisotropy* reflected by FICV (positively correlated to FA), and *orientation coherence* opposite to ODI (negatively correlated to FA). Our data underline that variations in FA values in peritumoral WM mostly depend on how FICV and ODI are affected by the tumor. Given the opposite influence of these two metrics on FA, when they vary in the same direction FA can result unaffected, as seen in the *Pattern A*, the most common pattern in our cohort (54.5% of patients). Although a typical FA decrease has been described in the nonenhancing peritumoral regions as more associated with subsequent tumor recurrence in glioblastoma (Bette et al., 2017), our analyses disclose that also areas with normal FA may hide pathological tissue, as identified by the NODDI metrics. Moreover, when ODI is greatly reduced, even a paradoxical increase of FA can arise in pathological WM, even in the presence of FICV and FECV alterations, as appreciable in the *Pattern B*. Surprisingly, this scenario may be rather common (36.4% of cases in our cohort). This phenomenon has already been described by voxel‐based analyses in cases of compressed fiber tracts, for example due to severe hydrocephalus (Radovnický, Adámek, Derner, & Sameš, 2016), and can be extremely misleading in diffusion data analysis, since high FA values are commonly interpreted as markers of WM health.

Only two patients showed different patterns (*Patterns C* and *D*), and even in such cases NODDI along‐tract metrics provided additional information with respect to DTI metrics, detecting a localized FISO increase.

The added value of along‐tract NODDI‐metrics was clear also when compared to MD, as MD was less sensitive than NODDI‐metrics overall, and showed a generalized tendency to increase aspecifically, regardless of the infiltrative or vasogenic origin of the edema.

The novelty of this work lies in precisely localizing the FA alteration along the tract, and in combining this information with the more comprehensive NODDI‐derived ones. Unraveling the reciprocal relations of microstructural diffusion metrics across corresponding tract sections may be pivotal to provide possible reasons for apparently ambiguous results. Finally, the meaning of the specific alterations in NODDI metrics in peritumoral WM still remains open to speculations. In fact, while the increase of extracellular volume was frequently found in gliomas and may be suggestive of peritumoral tissue infiltration from cancer cells (*Patterns A* and *B*), the circumscribed increase of free fluid is only detected in few patients and may be interpreted as indicative of a vasogenic edema component, as it is particularly evident in the single case of brain metastasis; this hypothesis is supported by recent findings from other studies (Kadota et al., 2020).

Nevertheless, histological validation of these hypotheses is necessary to correctly interpret NODDI‐metrics alterations and translate quantitative microstructural assessments into “virtual biopsies.”

### 4.3 Future perspectives and limitations

This study has some limitations. First, due to the relatively low number of healthy subjects enrolled, the NODDI‐metrics healthy microstructural profiles and their corresponding values should be considered an intrastudy reference rather than a standard reference database for future studies. In addition, age‐ and gender‐related variability of diffusion metrics were not accounted for, both when building the reference database and when performing patients' analysis, as the unique internal‐reference was compared to every patient regardless of the effect of such variability. Furthermore, the patient‐to‐controls comparison relies on the correspondence between the along‐tract vertices computed from the streamlines, and “damaged” fascicle models in tumor patients may affect the sampling of the vertices at the tract extremities, ultimately resulting in a shift between vertices in patients and controls. Nevertheless, we secured that this effect was minimal by selecting peritumoral tracts that were not excessively “damaged” by the tumor and by checking that the toolbox excluded the extremities of the fascicle models from the sampling (see Supplementary Figure 1). An additional limitation is that histopathological data regarding tumor infiltration along tracts were not available for our cohort; hence, only a better sensitivity of NODDI metrics in detecting along tract microstructural alterations could be proven, and potential NODDI false positive abnormal values could not be detected.

The methodology of along‐tract comparison of NODDI metrics validated in this study can serve as a model for future researches willing to assess along‐tract pathological modifications induced by other neurological or psychiatric conditions.

To our knowledge, previous along‐tract studies on WM neurodegeneration in Amyotrophic Lateral Sclerosis (ALS) (Sarica et al., 2017) and MS (Yeatman et al., 2018) only evaluated “classic” DTI metrics, possibly underestimating biologically specific alterations. At the same time, studies exploiting the NODDI model to detect WM anomalies in ALS (Broad et al., 2019), Parkinson's disease (Andica et al., 2018), unilateral cerebral palsy (Nemanich, Mueller, & Gillick, 2019), and stroke (Mastropietro et al., 2019) variously adopted a voxel‐wise or ROI‐based approach to compute the mean of NODDI metrics, possibly underestimating regionally specific alterations. Preliminary results in stroke patients demonstrated the superior specificity of NODDI in detecting subtle WM alterations when compared to FA (Adluru et al., 2014), while studies conducted on MS patients suggested a better detection of spinal cord lesions by means of NODDI rather than DTI‐based analysis (By, Xu, Box, Bagnato, & Smith, 2017).

Therefore, applying the along‐tract approach to extract the NODDI‐metrics could provide information about tract‐specific subtle anomalies that better reflects the microstructural status of WM tracts in such neurodegenerative conditions, potentially leading to the detection of tract‐specific pathological patterns at an earlier stage of diseases. Future studies following this approach could also improve the analysis by employing the Bingham‐NODDI model (Tariq, Schneider, Alexander, Gandini Wheeler‐Kingshott, & Zhang, 2016), in order to estimate anisotropic orientation dispersion that Watson‐NODDI cannot evaluate.

Furthermore, since the NODDI model was originally implemented to describe healthy cerebral tissue (Zhang et al., 2012), its application to define tumor‐induced WM alterations may lead to biased parameter estimates (Nilsson, Englund, Szczepankiewicz, van Westen, & Sundgren, 2018). However, it is important to highlight that our analyses did not focus on characterizing the tumor core, but rather on the anomalies of NODDI‐derived metrics in the peritumoral WM. Since the pre‐operative characterization of WM around the tumoral core still remains a clinical challenge, our study proposes a feasible approach that may unravel its microstructural composition. In this regard, further possible refinements may include the along‐tract estimation of additional quantitative indices, including VERDICT parameters (vascular, extracellular, and restricted diffusion for cytometry in tumors). The VERDICT model fits MRI data to complex biophysical models in order to estimate tumor‐specific microstructural features including cell radius and vascularization (Panagiotaki et al., 2014). VERDICT‐based along‐tract analyses, in particular, may help the characterization of tumor tissue infiltrating the WM tracts and may lead to a more biologically specific analysis.

## 5 CONCLUSION

The present work proposes the novel exploitation of along‐tract approach to extract NODDI‐derived diffusion metrics along the profile of WM tracts. The precise quantification of microstructural metrics along relevant fiber tracts of healthy controls and patients with brain tumors enhances the sensitivity of quantitative tractography both at a group and at a subject‐specific level. By incorporating the NODDI analysis into the structural and clinically relevant framework of the tract anatomy, this study highlights the higher accuracy of the FICV and ODI metrics in characterizing WM microstructural features with respect to “classic” DTI‐derived metrics, such as FA. The reference database of healthy controls' NODDI‐metrics, created as an internal standard for our analyses, is potentially useful to identify subtle deviations of pathological tract microstructural profiles from the healthy ones. Accordingly, our evaluations of patients with brain tumors allow to recognize pathological tract profiles, possibly providing quantitative signatures of the microstructural changes of peritumoral WM.

## CONFLICT OF INTEREST

The authors declare no conflict of interest.

## Supporting information


**Supplementary FIGURE 1** **. Schematic representation of fiber re‐parametrization**
Lookup table illustrating how each fascicle has been re‐parametrized in 100 points, starting from a common origin selected by an expert neuroradiologist. AF = Arcuate Fasciculus; FAT = Frontal Aslant Tract; IFOF = Inferior Fronto‐Occipital Fasciculus; UF = Uncinate Fasciculus; CING = Cingulum; CST = Corticospinal Tract; OR = Optic Radiation. Color‐coding illustrates the progression from point 1 (red) to point 100 (blue). Dotted lines represent the “skeletons” of tracts used as references for the cross‐sectional mean.
**Supplementary FIGURE 2‐ Normative reference of DTI and HARDI metrics along all tracts: AD, MD, RD**
Mean and 95% CI between the 15 healthy controls computed for each diffusion metric are displayed in line graphs: AD (orange), MD (purple), RD (pink). **A)** DTI‐derived metrics, extracted at b‐value 711 s/mm^2^; **B)** HARDI‐derived metrics, extracted at b‐value 3,000 s/mm^2^. It is evident that all metrics maintain very similar profiles at b = 700 and b = 3,000 s/mm^2^, but displaying higher absolute values at lower b‐values.
**Supplementary FIGURE 3‐ Inverse correlation between FICV and FECV**
Spearman's rank correlation coefficient (r) and p‐values (*P*) were computed for each fascicle, in order to analyze the relationship between FICV and FECV extracted from the tracts. These metrics display a statistically significant inverse correlation in all the WM tracts.
**Supplementary FIGURE 4‐ Inverse correlation between FA and ODI**
Spearman's rank correlation coefficient (r) and p‐values (*P*) were computed for each fascicle, in order to analyze the relationship between FA and ODI extracted from the tracts. These metrics display a strong statistical inverse correlation in all the WM tracts.
**Supplementary FIGURE 5‐ Weakly positive correlation between FA and FICV**
Spearman's rank correlation coefficient (r) and p‐values (*P*) were computed for each fascicle, in order to analyze the relationship between FA and FICV extracted from the tracts. These metrics display a statistically significant positive correlation in right UF, and in bilateral FATs, IFOFs, CINGs and CSTs. In all the other WM tracts, only a trend is observed.
**Supplementary FIGURE 6‐ Patients' cohort: three patterns of alterations in diffusivity metrics**
Along‐tract diffusion metrics of peritumoral tracts of all the 22 patients are shown individually. Patients' metrics are color‐coded, overlaid on the mean ± *SD* of reference metrics (gray). Divergences of more than ±2*SD* from “healthy microstructural profiles” are highlighted by colored asterisks (*) and black bars. Four different patterns of alterations in diffusivity were found in our cohort of patients with brain tumors. **A)** *Pattern A* shows normal FA along all the tract profile or along a consistent portion of it, in spite of consistently decreased FICV and increased FECV. **B)** *Pattern B* shows paradoxically increased FA along the tract, in spite of consistently decreased FICV and increased FECV. **C)** *Pattern C* identifies a FISO uspurge in the presence of completely abnormal FICV, FECV and FA. **D)** *Pattern D* identifies FISO as the only abnormal along‐tract diffusivity metric.
**Supplementary FIGURE 7‐ Patients' cohort: NODDI and MD diffusivity metrics**.
Along‐tract diffusion metrics of peritumoral tracts of all the 22 patients are shown individually. Patients' metrics are color‐ coded, overlaid on the mean ± *SD* of reference metrics (gray). Divergences of more than ±2*SD* from “healthy microstructural profiles” are highlighted by colored asterisks (*) and black bars. Both MD extracted at b = 711 s/mm^2^ and at b = 3,000 s/mm^2^ are shown. Irrespectively from the Patterns identified by the NODDI comparison with FA, pathological MD curves show nonspecific increases with respect to the reference values [Panels **A)**, **B)**, **C)**, and **D)**].

## Data Availability

The datasets for this study are available from the authors upon request.
