# Supplementary material for: Along‐tract statistics of neurite orientation dispersion and density imaging diffusion metrics to enhance MR tractography quantitative analysis in healthy controls and in patients with brain tumors
Source: Hum Brain Mapp. 2020 Dec 4;42(5):1268–86. doi: 10.1002/hbm.25291 (PMC7927309; doi:10.1002/hbm.25291)

Along-tract statistics of NODDI diffusion metrics to enhance MR tractography quantitative analysis in healthy controls and in patients with brain tumors

SUPPLEMENTARY MATERIAL: TABLES AND FIGURES

SUPPLEMENTARY TABLES

Supplementary Table 1 - ROI placement for each white matter fiber tract.
FA = Fractional Anisotropy; SMA = Supplementary Motor Area; LGN = Lateral Geniculate Nucleus.

White Matter Fiber Tracts	Seed-ROI	Target-ROI(s)	Further Precautions	Tractography or Anatomy References
**Arcuate Fascicle**	single-plane ROI, including corona radiata voxels with high anterior-posterior FA, in the coronal plane passing through the isthmus of corpus callosum	single-plane axial ROI including peritrigonal streamlines headed to the temporal lobe	supplemental no-ROIs to exclude streamlines headed to the insula	ROI placement as in Caverzasi et al (Caverzasi et al., 2015)
**Frontal Aslant Tract**	single-plane ROI including voxels corresponding to SMA, in the axial plane passing through the subcortical white matter of the superior frontal gyrus	single-plane sagittal ROI including streamlines headed to inferior frontal gyrus	None	ROI placement as in Sanvito et al (Sanvito et al., 2020)
**Inferior Fronto-Occipital Fascicle**	single-plane ROI including external capsule voxels with high anterior-posterior FA, in the coronal plane passing through the anterior commissure	single-plane coronal ROI including streamlines headed to the occipital lobe and superior parietal gyrus	supplemental no-ROIs to exclude streamlines belonging to the anterior commissure	ROI placement as in Caverzasi et al (Caverzasi et al., 2015; Caverzasi, Papinutto, Amirbekian, Berger, & Henry, 2014)
**Uncinate Fascicle**	single-plane ROI including external capsule voxels with high anterior-posterior FA, in the coronal plane passing through the anterior commissure	single-plane axial or coronal ROI including streamlines headed to the temporal pole	supplemental no-ROIs to exclude streamlines belonging to the anterior commissure	ROI placement as in Caverzasi et al (Caverzasi et al., 2015)
**Cingulum**	single-plane ROI including cingulum voxels (above corpus callosum) with high anterior-posterior FA, in the coronal plane passing through the isthmus of corpus callosum	None	supplemental no-ROIs to exclude streamlines belonging to corpus callosum	ROI placement based on CING trajectory as known from Wakana et al (Wakana, Jiang, Nagae-Poetscher, van Zijl, & Mori, 2004)
**Corticospinal Tract**	single-plane ROI including pons voxels with high cranio-caudal FA, in the axial plane passing through the middle cerebellar peduncles	single-plane axial (and sagittal) ROI(s) including streamlines headed to precentral gyrus	supplemental no-ROIs to exclude streamlines belonging to medial lemniscus	seed-ROI as in Yoo et al (Yoo et al., 2019); target-ROI as in Castellano et al (Castellano et al., 2012)
**Optic Radiation**	3D ROI of LGN was manually segmented on patient-specific 3D T1 images, registered to HARDI images, and then manually extended to include adjacent antero-lateral white matter, in particular OR voxels with high right-left FA	single-plane coronal ROI including voxels of sagittal stratum	supplemental no-ROIs to exclude streamlines belonging to IFOF or to optic tracts	LGN identification as in Kitajima et al (Kitajima et al., 2015); ROIs modified from Chamberland et al (Chamberland et al., 2017; Chamberland, Tax, & Jones, 2018)

Supplementary Table 2 - Statistical analyses of the normative reference NODDI-derived metrics and FA from the healthy controls. The ROUT method recognized outliers from each metric of each tract (Q= 1%). For each metric, outliers were identified also as the values over Q3 + 1.5*IQR (interquartile range), or under Q1 - 1.5* IQR. Median, Q1 and Q3 of the CV (coefficient of variation) are reported, for each metric of every tract. All the values are expressed in percentages (%).

	NODDI metrics & FA	Left Side					Right Side				
		% ROUT outliers	% IQR outliers	% CV			% ROUT outliers	% IQR outliers	% CV		
				Median	Q1	Q3			Median	Q1	Q3
Arcuate Fascicle	FICV	0,00	1,18	3,75	3,52	4,00	0,00	0,07	4,18	3,66	5,35
	FECV	0,42	2,71	9,48	8,38	10,88	0,00	1,81	11,10	10,04	12,59
	FISO	0,28	2,85	17,18	15,73	22,06	0,00	1,60	16,08	14,17	20,64
	ODI	0,28	1,39	9,11	7,76	10,76	0,97	3,06	8,56	7,28	10,25
	FA	0,76	2,50	9,38	8,39	10,36	0,76	2,71	8,59	8,07	9,37
Frontal Aslant Tract	FICV	0,00	0,00	5,28	4,66	5,85	0,35	2,64	5,36	4,83	6,78
	FECV	0,00	0,21	11,28	10,28	12,87	0,00	0,76	10,84	9,58	12,52
	FISO	0,28	3,13	22,14	17,25	31,88	0,00	1,94	21,42	16,86	32,79
	ODI	0,76	1,53	13,25	12,54	18,15	1,11	3,33	13,63	10,43	17,53
	FA	0,28	1,53	12,31	10,82	18,13	0,97	2,71	13,10	11,15	16,95
IFOF	FICV	0,00	1,53	6,25	5,65	6,69	0,28	2,15	4,91	4,62	6,42
	FECV	0,69	2,29	9,50	8,34	10,88	0,00	0,56	7,79	7,04	8,68
	FISO	1,46	3,33	40,28	34,18	54,32	0,76	3,26	35,07	26,43	46,65
	ODI	2,57	3,19	11,24	9,50	13,79	1,18	2,92	9,58	8,28	10,92
	FA	0,42	0,69	14,67	12,58	17,58	0,56	1,74	13,97	10,93	17,61
Uncinate Fascicle	FICV	0,00	2,15	8,94	7,61	10,77	0,56	1,67	9,45	6,16	10,67
	FECV	0,21	0,49	11,12	9,15	12,16	0,00	1,46	9,08	6,80	11,45
	FISO	1,11	2,64	66,13	56,24	73,71	0,35	3,06	61,93	58,63	66,59
	ODI	0,00	0,07	12,45	8,80	15,93	0,00	0,07	16,99	15,24	20,55
	FA	0,00	2,15	14,06	12,91	17,25	0,00	0,83	16,54	15,13	18,81
Cingulum	FICV	0,00	0,14	8,94	7,61	9,38	0,00	0,00	7,08	6,89	7,29
	FECV	0,00	0,76	9,83	7,92	12,04	0,00	0,56	9,80	9,14	10,80
	FISO	0,00	0,69	37,56	24,40	46,26	0,00	0,83	36,98	33,65	40,81
	ODI	0,00	0,56	14,81	11,45	20,17	0,00	2,57	11,30	9,39	14,62
	FA	0,21	2,43	15,13	13,96	17,58	0,00	1,88	10,81	9,90	11,62
Cortico-spinal Tract	FICV	0,14	1,53	6,40	4,22	9,43	0,14	1,88	4,78	3,89	9,20
	FECV	0,07	0,97	20,07	13,86	28,78	0,21	3,26	15,39	13,09	25,79
	FISO	0,56	2,15	29,26	25,47	35,32	0,14	1,67	24,64	17,22	36,54
	ODI	0,49	1,04	12,79	11,23	14,83	1,04	2,78	13,38	11,12	15,76
	FA	0,42	0,56	14,53	13,46	15,59	0,14	2,71	11,81	10,43	14,10
Optic Radiation	FICV	0,35	1,94	7,00	6,49	8,23	0,00	1,53	7,14	6,77	7,98
	FECV	0,14	0,83	11,84	10,49	13,53	0,00	0,90	9,52	7,71	12,86
	FISO	1,53	2,99	35,38	30,93	52,45	0,07	1,60	30,82	26,53	34,08
	ODI	0,56	2,64	12,70	9,49	15,66	0,35	2,43	14,79	11,54	18,82
	FA	0,63	1,46	17,20	14,36	18,44	0,07	0,35	16,29	14,47	19,72

Supplementary Table 3 - Descriptive statistics of the normative reference DTI- and HARDI-derived metrics extracted along seven WM tracts of the healthy controls, bilaterally (Supplementary Tables 2A and 2B, respectively). For each of the 96 points along the tracts, a cross-sectional mean from each subject was extracted, and SD and 95% CI width of the means from all subjects were calculated. This table reports minimum and maximum values of SD and 95% CI width.

	A)	Left Side				Right Side			
	**Normative DTI metrics**	**Min SD**	**Max SD**	**Min 95% CI width**	**Max 95% CI width**	**Min SD**	**Max SD**	**Min 95% CI width**	**Max 95% CI width**
**Arcuate Fascicle**	**DTI_FA**	0,0250	0,0561	0,0276	0,0622	0,0159	0,0564	0,0176	0,0624
	**DTI_AD**	0,0247	0,0677	0,0260	0,0740	0,0290	0,0659	0,0340	0,0740
	**DTI_MD**	0,0151	0,0435	0,0166	0,0482	0,0125	0,0496	0,0138	0,0550
	**DTI_RD**	0,0207	0,0485	0,0230	0,0536	0,0206	0,0521	0,0228	0,0578
**Frontal Aslant**	**DTI_FA**	0,0221	0,0868	0,0244	0,0962	0,0113	0,0874	0,0126	0,0968
	**DTI_AD**	0,0351	0,1484	0,0400	0,1640	0,0298	0,1373	0,0330	0,1540
	**DTI_MD**	0,0165	0,1502	0,0182	0,1660	0,0228	0,1420	0,0252	0,1560
	**DTI_RD**	0,0351	0,1518	0,0390	0,1680	0,0294	0,1446	0,0326	0,1600
**IFOF**	**DTI_FA**	0,0202	0,0566	0,0222	0,0628	0,0157	0,0457	0,0172	0,0508
	**DTI_AD**	0,0265	0,0967	0,0300	0,1080	0,0269	0,1159	0,0300	0,1280
	**DTI_MD**	0,0312	0,0933	0,0346	0,1034	0,0240	0,1125	0,0266	0,1246
	**DTI_RD**	0,0307	0,1027	0,0340	0,1138	0,0292	0,1192	0,0322	0,1320
**Uncinate Fascicle**	**DTI_FA**	0,0309	0,0809	0,0342	0,0896	0,0276	0,1070	0,0306	0,1186
	**DTI_AD**	0,0426	0,1591	0,0480	0,1780	0,0429	0,1316	0,0480	0,1460
	**DTI_MD**	0,0391	0,0991	0,0434	0,1102	0,0380	0,1259	0,0422	0,1398
	**DTI_RD**	0,0426	0,1165	0,0472	0,1290	0,0404	0,1244	0,0448	0,1378
**Cingulum**	**DTI_FA**	0,0229	0,0971	0,0254	0,1076	0,0293	0,0658	0,0324	0,0728
	**DTI_AD**	0,0362	0,0923	0,0400	0,1020	0,0365	0,0797	0,0400	0,0880
	**DTI_MD**	0,0451	0,0831	0,0500	0,0920	0,0325	0,0601	0,0360	0,0666
	**DTI_RD**	0,0574	0,1137	0,0636	0,1258	0,0378	0,0761	0,0418	0,0842
**Cortico-spinal**	**DTI_FA**	0,0431	0,0904	0,0478	0,1002	0,0375	0,0956	0,0416	0,1060
	**DTI_AD**	0,0446	0,2911	0,0480	0,3220	0,0283	0,3838	0,0320	0,4260
	**DTI_MD**	0,0215	0,2619	0,0238	0,2900	0,0173	0,3751	0,0192	0,4160
	**DTI_RD**	0,0340	0,2556	0,0378	0,2840	0,0275	0,3762	0,0306	0,4160
**Optic Radiation**	**DTI_FA**	0,0235	0,0929	0,0260	0,1030	0,0303	0,0743	0,0336	0,0824
	**DTI_AD**	0,0522	0,3027	0,0560	0,3340	0,0502	0,1901	0,0560	0,2120
	**DTI_MD**	0,0286	0,2790	0,0318	0,3080	0,0296	0,1724	0,0328	0,1900
	**DTI_RD**	0,0259	0,2685	0,0288	0,2960	0,0374	0,1654	0,0414	0,1826

B) Normative HARDI metrics		Left Side Min SD	Left Side Max SD	Left Side Min 95% CI width	Left Side Max 95% CI width	Right Side Min SD	Right Side Max SD	Right Side Min 95% CI width	Right Side Max 95% CI width
Arcuate Fascicle	HARDI_FA	0,0200	0,0750	0,0220	0,0830	0,0206	0,0577	0,0228	0,0638
	HARDI_AD	0,0234	0,0574	0,0258	0,0636	0,0227	0,0520	0,0252	0,0576
	HARDI_MD	0,0082	0,0291	0,0092	0,0322	0,0106	0,0281	0,0118	0,0310
	HARDI_RD	0,0114	0,0364	0,0126	0,0404	0,0094	0,0326	0,0104	0,0360
Frontal Aslant Tract	HARDI_FA	0,0142	0,0857	0,0158	0,0948	0,0200	0,0999	0,0222	0,1106
	HARDI_AD	0,0213	0,0679	0,0236	0,0752	0,0203	0,1006	0,0224	0,1114
	HARDI_MD	0,0114	0,0415	0,0126	0,0460	0,0117	0,0351	0,0128	0,0388
	HARDI_RD	0,0182	0,0399	0,0200	0,0442	0,0150	0,0389	0,0166	0,0430
IFOF	HARDI_FA	0,0280	0,0969	0,0310	0,1074	0,0113	0,0902	0,0124	0,0998
	HARDI_AD	0,0153	0,0897	0,0170	0,0994	0,0219	0,0821	0,0244	0,0910
	HARDI_MD	0,0135	0,0496	0,0150	0,0550	0,0113	0,0530	0,0126	0,0588
	HARDI_RD	0,0164	0,0565	0,0182	0,0626	0,0168	0,0747	0,0186	0,0828
Uncinate Fascicle	HARDI_FA	0,0187	0,0855	0,0206	0,0946	0,0322	0,0784	0,0356	0,0868
	HARDI_AD	0,0188	0,0720	0,0210	0,0798	0,0314	0,0695	0,0348	0,0770
	HARDI_MD	0,0197	0,0456	0,0218	0,0504	0,0231	0,0559	0,0256	0,0620
	HARDI_RD	0,0252	0,0553	0,0280	0,0612	0,0237	0,0599	0,0262	0,0664
Cingulum	HARDI_FA	0,0293	0,0813	0,0324	0,0900	0,0239	0,0520	0,0264	0,0576
	HARDI_AD	0,0242	0,0597	0,0268	0,0660	0,0224	0,0467	0,0248	0,0518
	HARDI_MD	0,0291	0,0409	0,0322	0,0454	0,0238	0,0334	0,0264	0,0372
	HARDI_RD	0,0347	0,0585	0,0384	0,0648	0,0275	0,0437	0,0306	0,0484
Cortico-spinal Tract	HARDI_FA	0,0286	0,1159	0,0316	0,1284	0,0310	0,1015	0,0344	0,1124
	HARDI_AD	0,0328	0,1016	0,0364	0,1126	0,0299	0,1199	0,0330	0,1328
	HARDI_MD	0,0097	0,0814	0,0108	0,0900	0,0114	0,0911	0,0126	0,1008
	HARDI_RD	0,0154	0,1063	0,0170	0,1178	0,0113	0,0802	0,0126	0,0888
Optic Radiation	HARDI_FA	0,0411	0,0969	0,0454	0,1074	0,0301	0,0992	0,0334	0,1098
	HARDI_AD	0,0270	0,1122	0,0298	0,1244	0,0367	0,1134	0,0408	0,1250
	HARDI_MD	0,0225	0,1022	0,0250	0,1132	0,0211	0,1475	0,0232	0,1634
	HARDI_RD	0,0183	0,1247	0,0204	0,1382	0,0234	0,1763	0,0260	0,1952

e.g. **left AF FA: Mean & SD** **left AF FA: Mean & 95% CI**

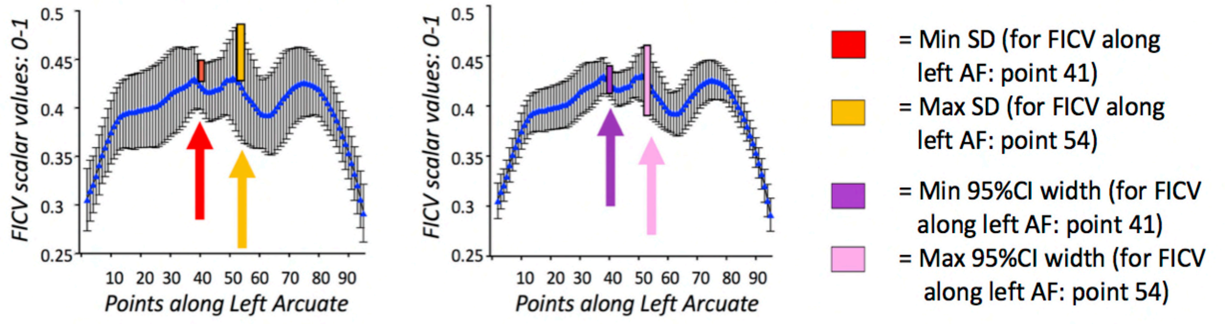

SUPPLEMENTARY FIGURES

Supplementary Figure 1- Schematic representation of fiber re-parametrization
Lookup table illustrating how each fascicle has been re-parametrized in 100 points, starting from a common origin selected by an expert neuroradiologist.
AF= Arcuate Fasciculus; FAT= Frontal Aslant Tract; IFOF= Inferior Fronto-Occipital Fasciculus; UF= Uncinate Fasciculus; CING= Cingulum; CST= Corticospinal Tract; OR= Optic Radiation. Color-coding illustrates the progression from point 1 (red) to point 100 (blue). Dotted lines represent the 'skeletons' of tracts used as references for the cross-sectional mean.

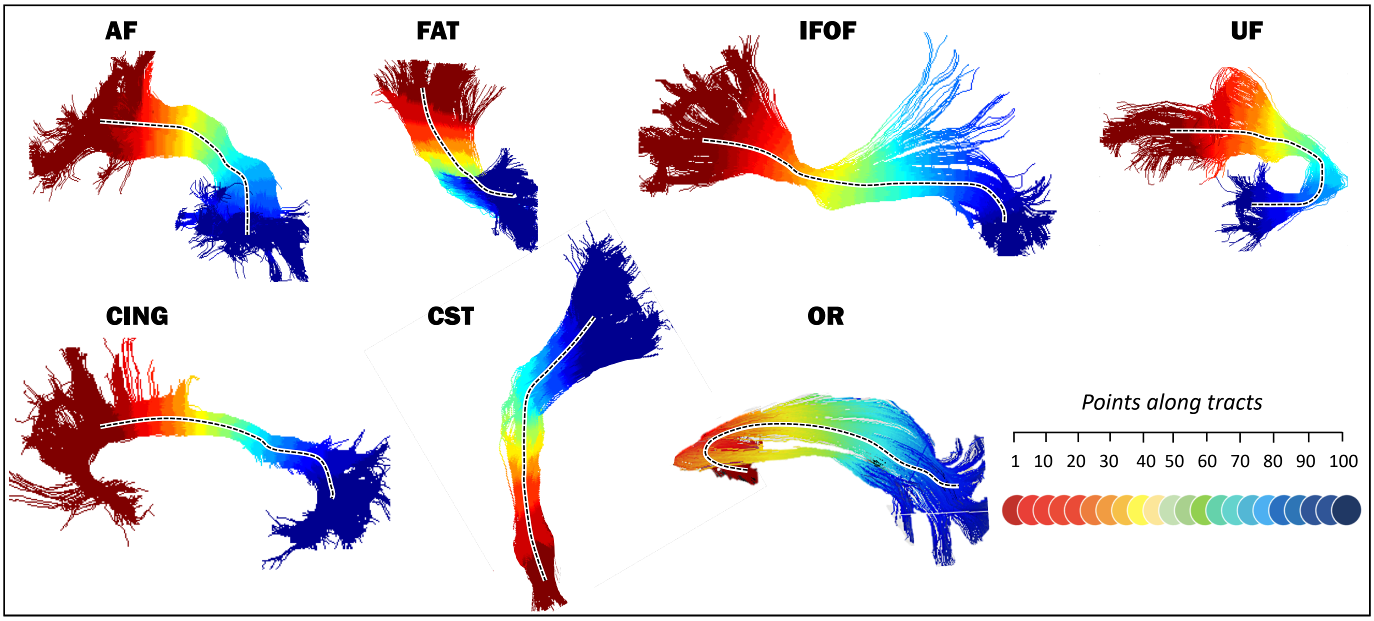

Supplementary Figure 2- Normative reference of DTI and HARDI metrics along all tracts: AD, MD, RD

Mean and 95% CI between the 15 healthy controls computed for each diffusion metric are displayed in line graphs: AD (orange), MD (purple), RD (pink).

A) DTI-derived metrics, extracted at b-value 711 s/mm^2; **B)** HARDI-derived metrics, extracted at b-value 3000 s/mm^2. It is evident that all metrics maintain very similar profiles at b=700 and b=3000 s/mm^2, but displaying higher absolute values at lower b-values.

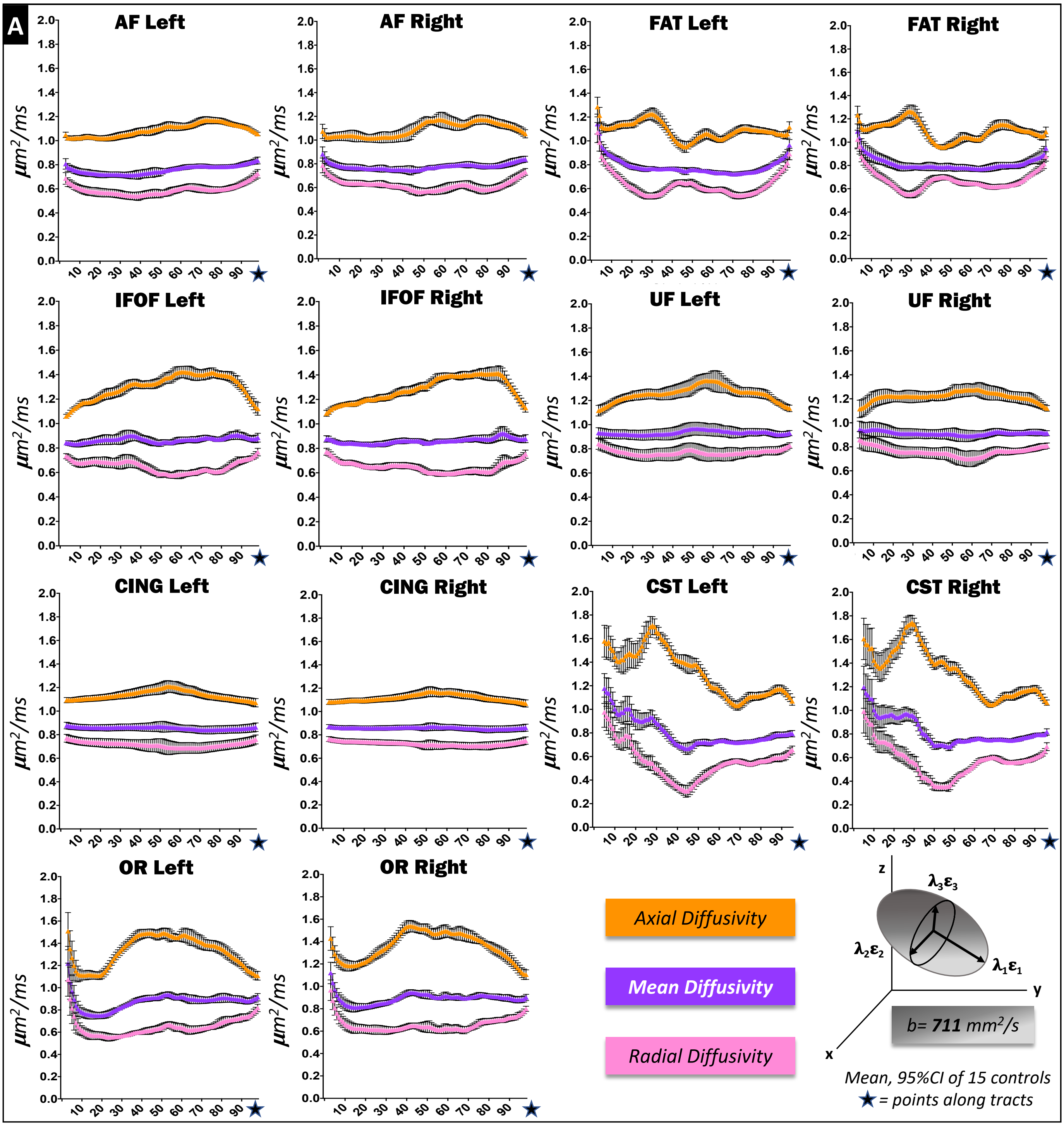

Supplementary Figure 2- Normative reference of DTI and HARDI metrics along all tracts: AD, MD, RD

Mean and 95% CI between the 15 healthy controls computed for each diffusion metric are displayed in line graphs: AD (orange), MD (purple), RD (pink).

A) DTI-derived metrics, extracted at b-value 711 s/mm^2; **B)** HARDI-derived metrics, extracted at b-value 3000 s/mm^2. It is evident that all metrics maintain very similar profiles at b=700 and b=3000 s/mm^2, but displaying higher absolute values at lower b-values.

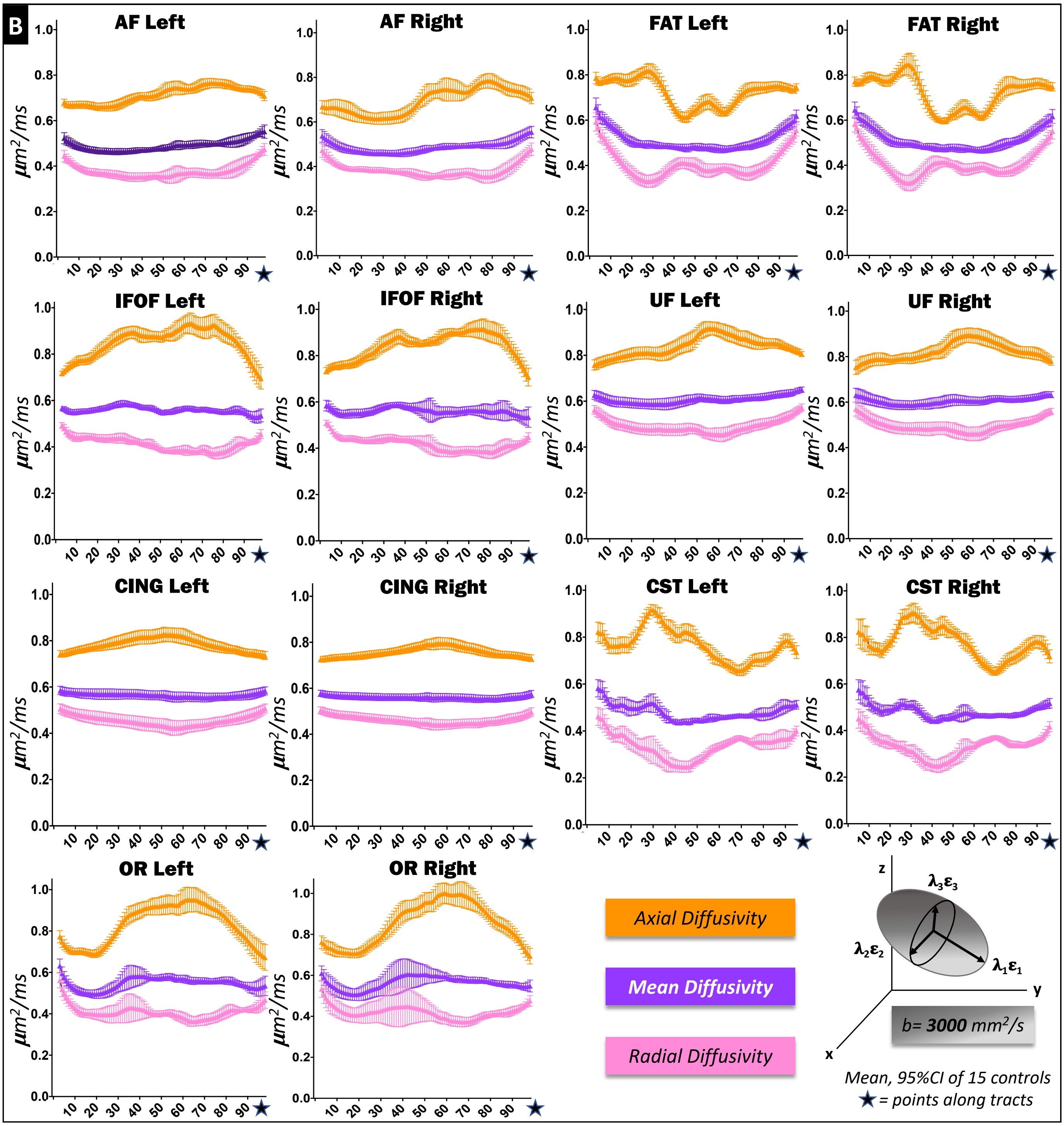

Supplementary Figure 3- Inverse correlation between FICV and FECV

Spearman's rank correlation coefficient (r) and p-values (*P*) were computed for each fascicle, in order to analyze the relationship between FICV and FECV extracted from the tracts. These metrics display a statistically significant inverse correlation in all the WM tracts.

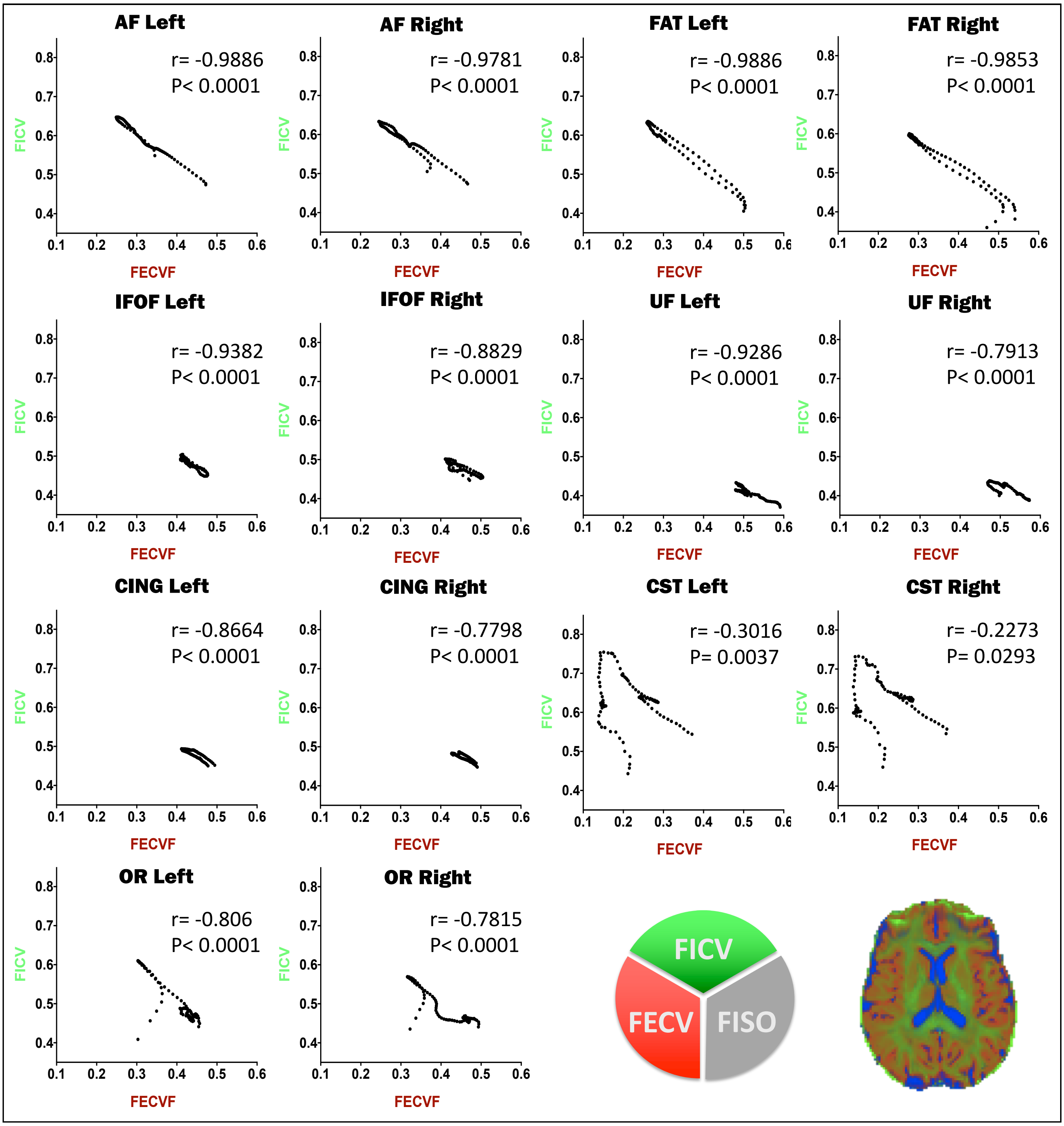

Supplementary Figure 4- Inverse correlation between FA and ODI
Spearman's rank correlation coefficient (r) and p-values (*P*) were computed for each fascicle, in order to analyze the relationship between FA (b=3000 s/mm^2) and ODI extracted from the tracts. These metrics display a strong statistical inverse correlation in all the WM tracts.

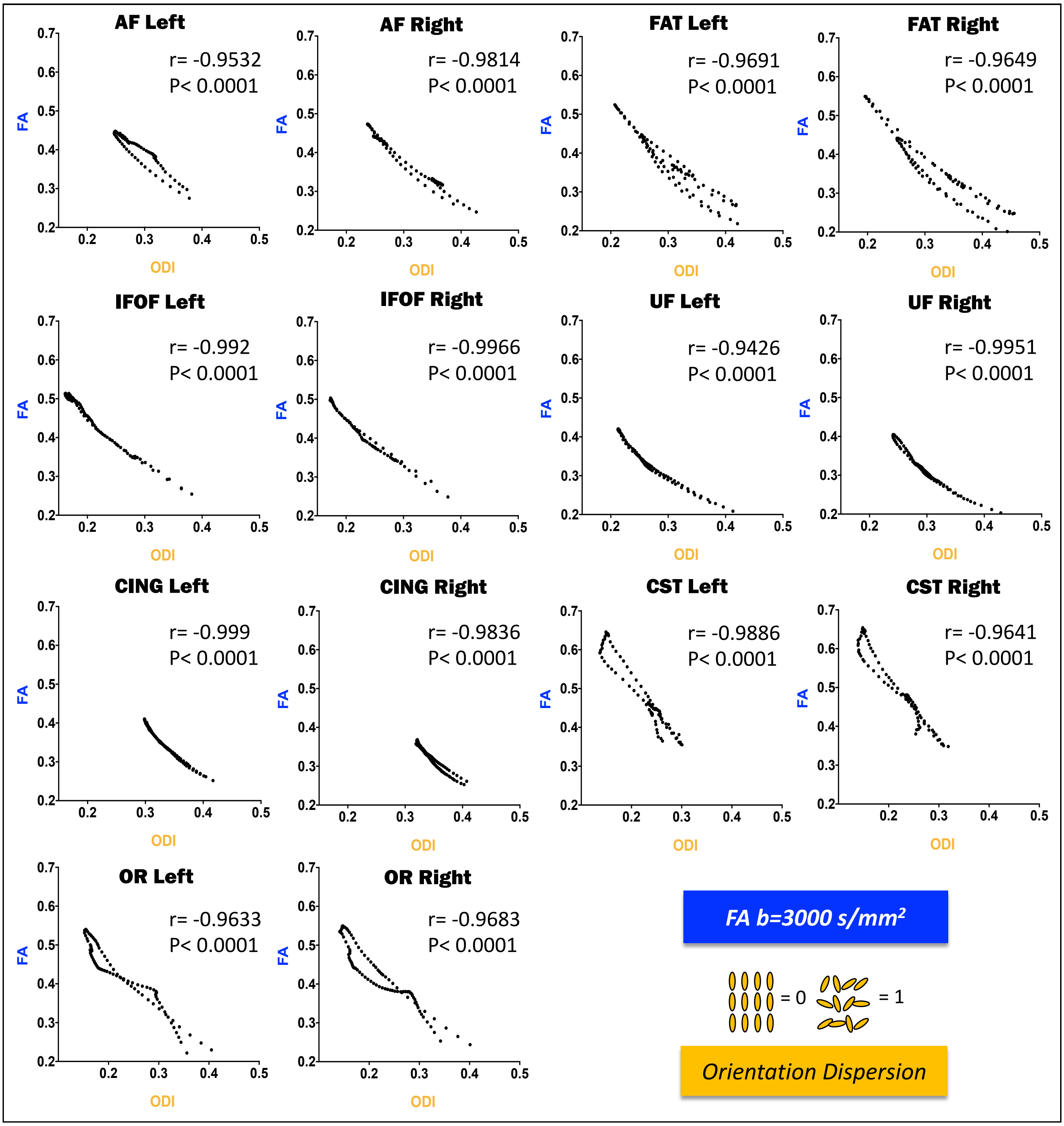

Supplementary Figure 5- Weakly positive correlation between FA and FICV

Spearman's rank correlation coefficient (r) and p-values (*P*) were computed for each fascicle, in order to analyze the relationship between FA (b=3000 s/mm²) and FICV extracted from the tracts. These metrics display a statistically significant positive correlation in right UF, and in bilateral FATs, IFOFs, CINGs and CSTs. In all the other WM tracts, only a trend is observed.

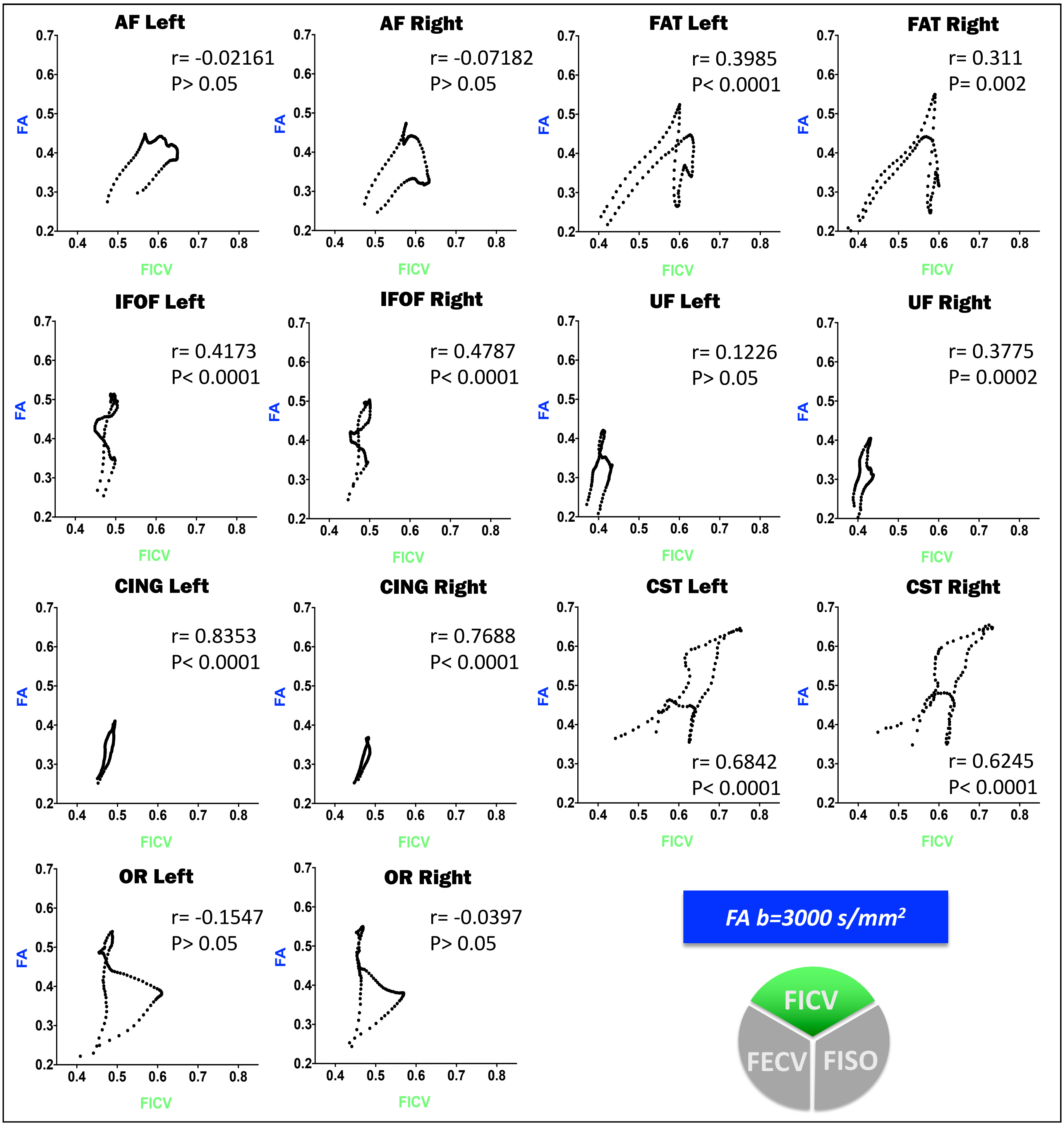

Supplementary Figure 6- Patients' cohort: three patterns of alterations in diffusivity metrics .
Along-tract diffusion metrics of peritumoral tracts of all the 22 patients are shown individually. Patients' metrics are color-coded, overlaid on the mean ± SD of reference metrics (grey). Divergences of more than ± 2SD from 'healthy microstructural profiles' are highlighted by colored asterisks (*) and black bars. Four different patterns of alterations in diffusivity were found in our cohort of patients with brain tumors. FA extracted at b=3000 s/mm^2 is shown. **A)** *Pattern A* shows normal FA along all the tract profile or along a consistent portion of it, in spite of consistently decreased FICV and increased FECV. **B)** *Pattern B* shows paradoxically increased FA along the tract, in spite of consistently decreased FICV and increased FECV. **C)** *Pattern C* identifies a FISO uspurge in the presence of completely abnormal FICV, FECV and FA. **D)** *Pattern D* identifies FISO as the only abnormal along-tract diffusivity metric.

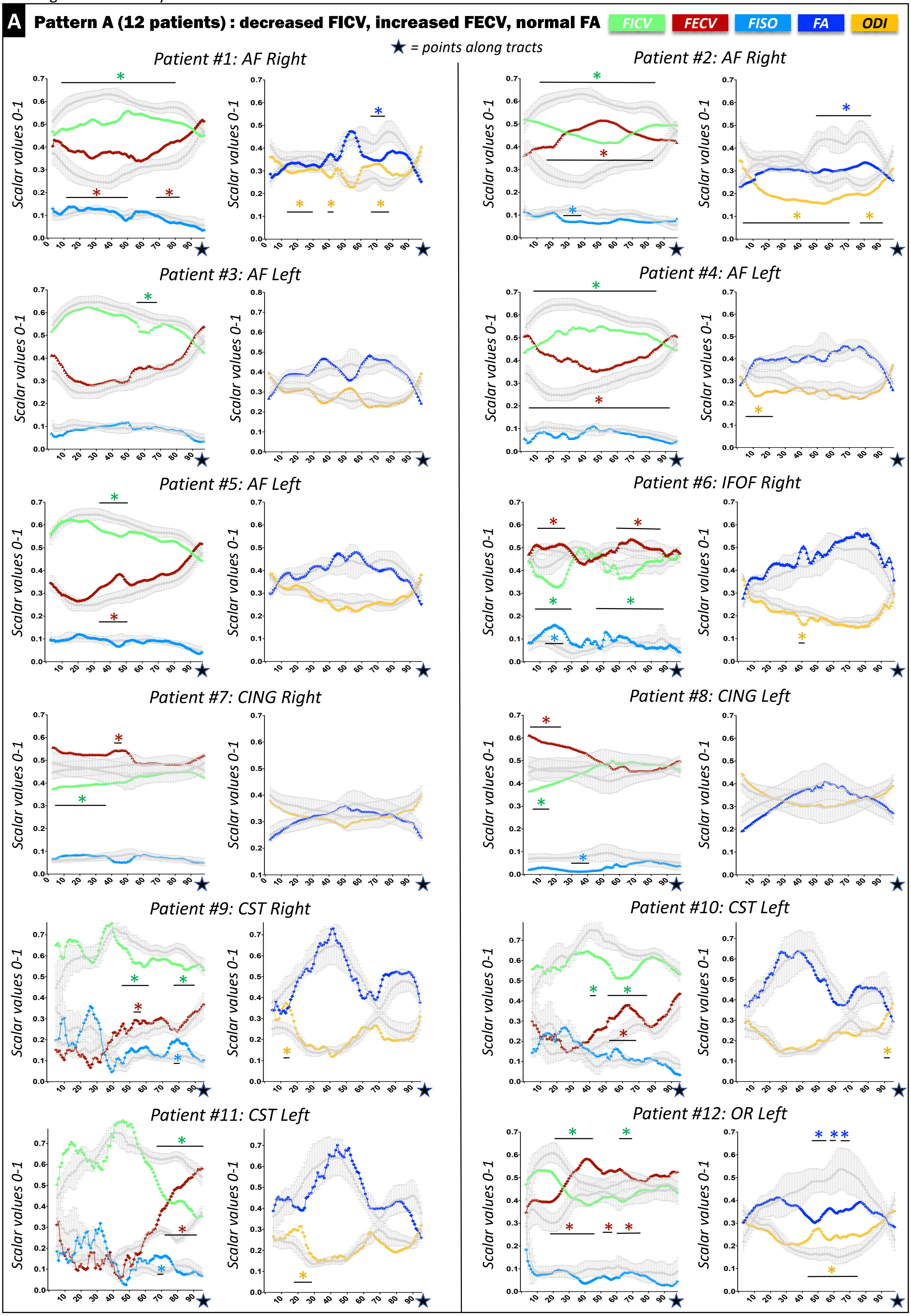

Supplementary Figure 6- Patients' cohort: three patterns of alterations in diffusivity metrics .

Along-tract diffusion metrics of peritumoral tracts of all the 22 patients are shown individually. Patients' metrics are color-coded, overlaid on the mean ± SD of reference metrics (grey). Divergences of more than ± 2SD from 'healthy microstructural profiles' are highlighted by colored asterisks (*) and black bars. Four different patterns of alterations in diffusivity were found in our cohort of patients with brain tumors. FA extracted at b=3000 s/mm^2 is shown. **A)** *Pattern A* shows normal FA along all the tract profile or along a consistent portion of it, in spite of consistently decreased FICV and increased FECV. **B)** *Pattern B* shows paradoxically increased FA along the tract, in spite of consistently decreased FICV and increased FECV. **C)** *Pattern C* identifies a FISO uspurge in the presence of completely abnormal FICV, FECV and FA. **D)** *Pattern D* identifies FISO as the only abnormal along-tract diffusivity metric.

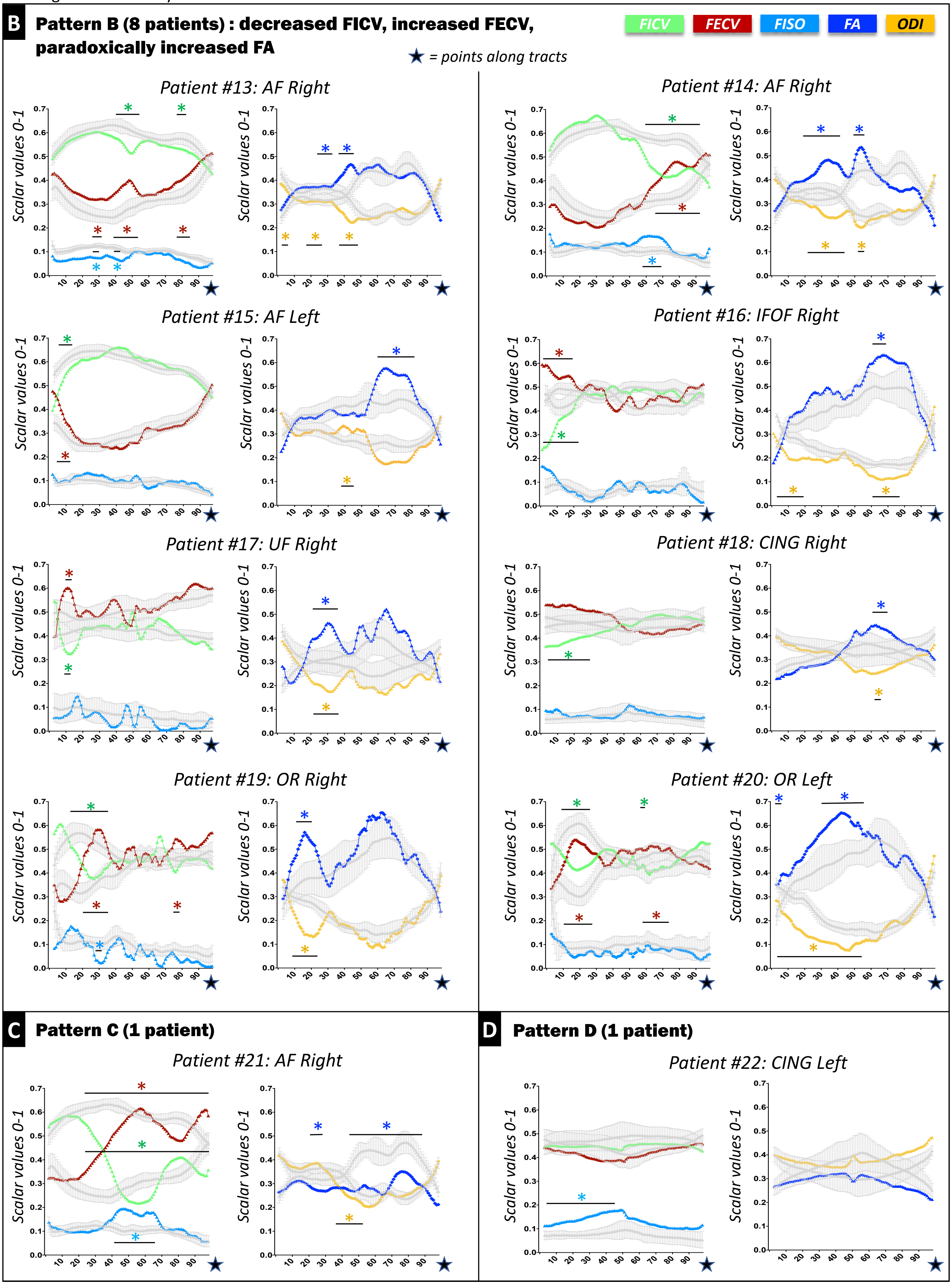

Supplementary Figure 7- Patients' cohort: NODDI and MD diffusivity metrics.
Along-tract diffusion metrics of peritumoral tracts of all the 22 patients are shown individually. Patients' metrics are color-coded, overlaid on the mean ± SD of reference metrics (grey). Divergences of more than ± 2SD from 'healthy microstructural profiles' are highlighted by colored asterisks (*) and black bars. Both MD extracted at b=711 s/mm^2 and at b=3000 s/mm^2 are shown. Irrespectively from the Patterns identified by the NODDI comparison with FA, pathological MD curves show non-specific increases with respect to the reference values [Panels **A)**, **B)**, **C)**, and **D)**].

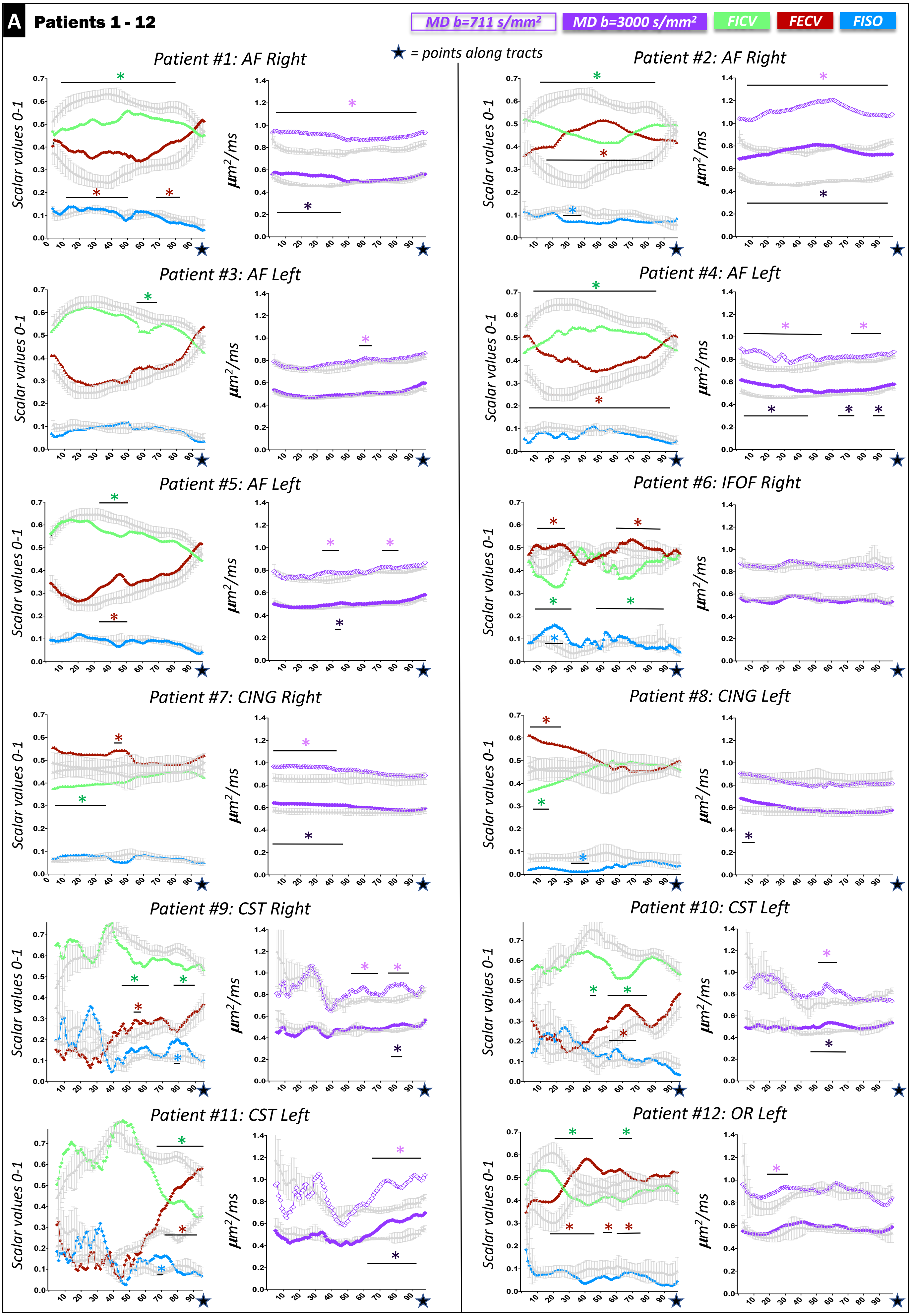

Supplementary Figure 7- Patients' cohort: NODDI and MD diffusivity metrics.
Along-tract diffusion metrics of peritumoral tracts of all the 22 patients are shown individually. Patients' metrics are color-coded, overlaid on the mean ± SD of reference metrics (grey). Divergences of more than ± 2SD from 'healthy microstructural profiles' are highlighted by colored asterisks (*) and black bars. Both MD extracted at b=711 s/mm^2 and at b=3000 s/mm^2 are shown. Irrespectively from the Patterns identified by the NODDI comparison with FA, pathological MD curves show non-specific increases with respect to the reference values [Panels **A)**, **B)**, **C)**, and **D)**].

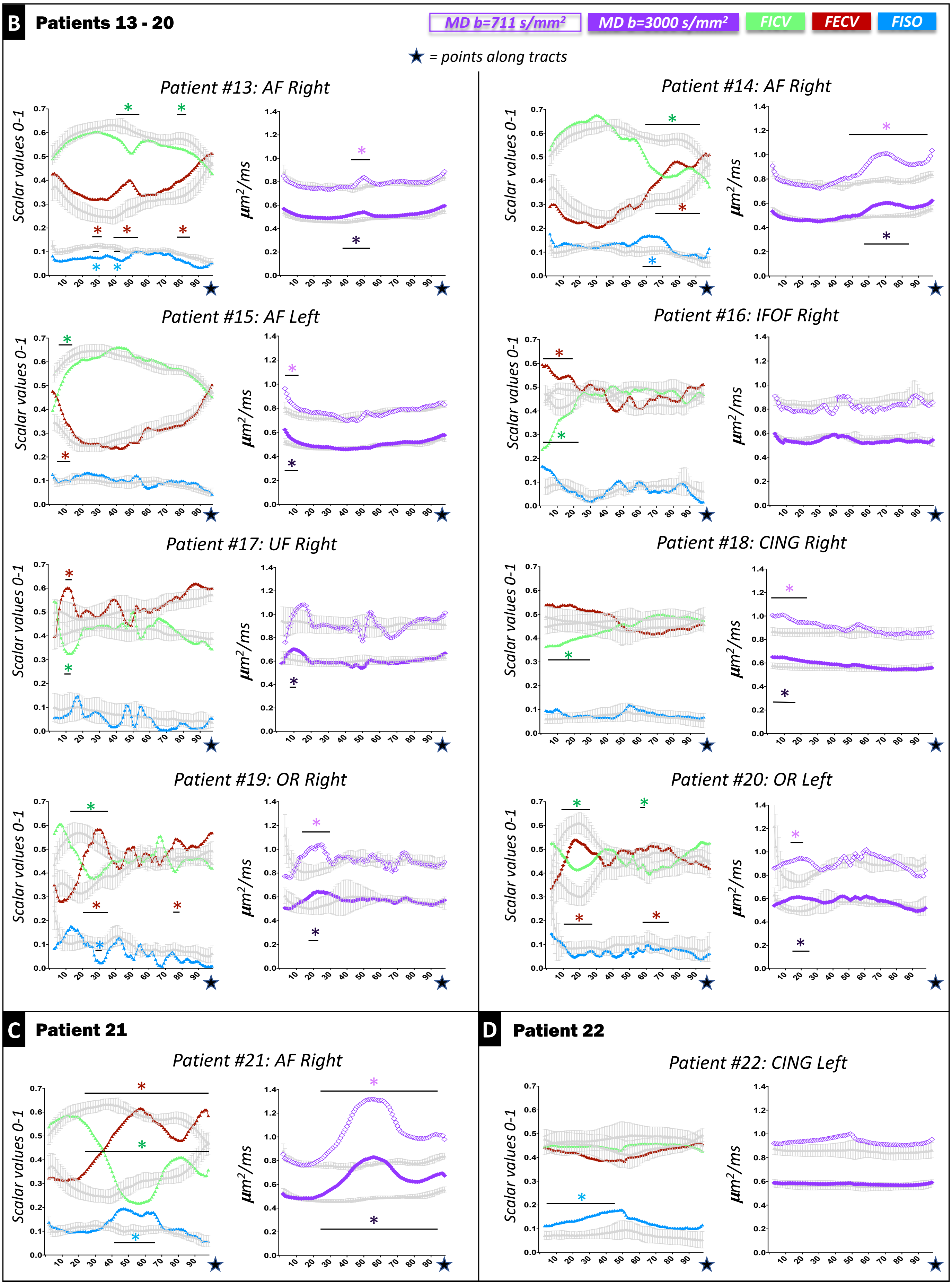

Supplement: Supplementary file 1 — Supplementary FIGURE 1 . Schematic representation of fiber re‐parametrization Lookup table illustrating how each fascicle has been re‐parametrized in 100 points, starting from a common origin selected by an expert neuroradiologist. AF = Arcuate Fasciculus; FAT = Frontal Aslant Tract; IFOF = Inferior Fronto‐Occipital Fasciculus; UF = Uncinate Fasciculus; CING = Cingulum; CST = Corticospinal Tract; OR = Optic Radiation. Color‐coding illustrates the progression from point 1 (red) to point 100 (blue). Dotted lines represent the “skeletons” of tracts used as references for the cross‐sectional mean. Supplementary FIGURE 2‐ Normative reference of DTI and HARDI metrics along all tracts: AD, MD, RD Mean and 95% CI between the 15 healthy controls computed for each diffusion metric are displayed in line graphs: AD (orange), MD (purple), RD (pink). A) DTI‐derived metrics, extracted at b‐value 711 s/mm2; B) HARDI‐derived metrics, extracted at b‐value 3,000 s/mm2. It is evident that all metrics maintain very similar profiles at b = 700 and b = 3,000 s/mm2, but displaying higher absolute values at lower b‐values. Supplementary FIGURE 3‐ Inverse correlation between FICV and FECV Spearman's rank correlation coefficient (r) and p‐values (P) were computed for each fascicle, in order to analyze the relationship between FICV and FECV extracted from the tracts. These metrics display a statistically significant inverse correlation in all the WM tracts. Supplementary FIGURE 4‐ Inverse correlation between FA and ODI Spearman's rank correlation coefficient (r) and p‐values (P) were computed for each fascicle, in order to analyze the relationship between FA and ODI extracted from the tracts. These metrics display a strong statistical inverse correlation in all the WM tracts. Supplementary FIGURE 5‐ Weakly positive correlation between FA and FICV Spearman's rank correlation coefficient (r) and p‐values (P) were computed for each fascicle, in order to analyze the relationship between FA and FIC [file HBM-42-1268-s001.pdf]
